# Oleyl Conjugated Histidine-Arginine Cell-Penetrating Peptides as Promising Agents for siRNA Delivery

**DOI:** 10.3390/pharmaceutics14040881

**Published:** 2022-04-18

**Authors:** Muhammad Imran Sajid, Dindyal Mandal, Naglaa Salem El-Sayed, Sandeep Lohan, Jonathan Moreno, Rakesh Kumar Tiwari

**Affiliations:** 1 Center for Targeted Drug Delivery, Department of Biomedical and Pharmaceutical Sciences, Chapman University School of Pharmacy, Harry and Diane Rinker Health Science Campus, Irvine, CA 92618, USA; sajid@chapman.edu (M.I.S.); dmandal@kiitbiotech.ac.in (D.M.); nibrahim@chapman.edu (N.S.E.-S.); lohan@chapman.edu (S.L.); jonmoreno@chapman.edu (J.M.); 2 Faculty of Pharmacy, University of Central Punjab, Lahore 54000, Pakistan; 3 School of Biotechnology, KIIT Deemed to Be University, Bhubaneswar 751024, India; 4 Cellulose and Paper Department, National Research Center, Dokki, Cairo 12622, Egypt

**Keywords:** siRNA, STAT-3, cell-penetrating peptides, siRNA delivery, RNA interference, Western blotting

## Abstract

Recent approvals of siRNA-based products motivated the scientific community to explore siRNA as a treatment option for several intractable ailments, especially cancer. The success of approved siRNA therapy requires a suitable and safer drug delivery agent. Herein, we report a series of oleyl conjugated histidine–arginine peptides as a promising nonviral siRNA delivery tool. The conjugated peptides were found to bind with the siRNA at N/P ratio ≥ 2 and demonstrated complete protection for the siRNA from early enzymatic degradation at N/P ratio ≥ 20. Oleyl-conjugated peptide -siRNA complexes were found to be noncytotoxic in breast cancer cells (MCF-7 and MDA-MB-231) and normal breast epithelial cells (MCF 10A) at N/P ratio of ~40. The oleyl-R_3_-(HR)_4_ and oleyl-R_4_-(HR)_4_ showed ~80-fold increased cellular uptake in MDA-MB-231 cells at N/P 40. Moreover, the conjugated peptides-siRNA complexes form nanocomplexes (~115 nm in size) and have an appropriate surface charge to interact with the cell membrane and cause cellular internalization. Furthermore, this study provides a proof-of-concept that oleyl-R_5_-(HR)_4_ can efficiently silence STAT-3 gene (~80% inhibition) in MDA-MB-231 cells with similar effectiveness to Lipofectamine. Further exploration of this approach holds a great promise in discovering a successful in vivo siRNA delivery agent with a favorable pharmacokinetic profile.

## 1. Introduction

In 1998, Andrew Fire and Craig Mellow published a seminal paper in which they discovered the phenomenon of post-transcriptional gene silencing (PTGS) and termed it as RNA interference (RNAi) [1]. Later, in 2001, two research groups reported that 21 to 22 nucleotide (nt) double-stranded RNAs, popularly known as small interfering RNAs (siRNAs), can induce gene silencing in mammalian cells without causing nonspecific interferon response [2,3]. These siRNAs later kicked off a revolution in biology due to their potential to inhibit virtually all genes by a base sequence alone, which gives RNAi several advantages over small-molecule drugs as a therapeutic strategy [4]. Approval of the first siRNA-based drug Patisiran (Onpattro^TM^) by the U.S. Food and Drug Administration (FDA) in 2018, followed by approvals of Givosiran (Givlaari™), and lumasiran (OXLUMO™) has fostered a renewed interest in siRNA-based therapeutics [4].

However, there are several extracellular as well as intracellular challenges related to the effectiveness of siRNA therapy, which include its membrane impermeability degradation by endonucleases and RNAases, clearance of siRNA delivery system by the reticuloendothelial system, endosomal entrapment and off-target RNAi activity [5]. Several delivery systems and transfection agents have been investigated to address these challenges. For example, lipofectamine [6], polyethyl imine (PEI) [7], a dynamic polyconjugate system (DPC) [8], magnetic and metallic nanoparticles [6], viral carriers [9], dendrimers [10,11], and liposome-based approaches [12,13] have been studied extensively for intracellular delivery of siRNA. Most of these delivery systems have their advantages as well as limitations [14,15,16,17]. The approved siRNA products employed solid lipid nanoparticles as delivery vectors [18], and these showed adequate safety in both in vitro and in vivo studies; nevertheless, there are reports that raise concern for their long-term use [19,20]. Therefore, there is a critical need to develop non-toxic and more efficient siRNA delivery tools. 

Over the past two decades, it has been found that cell-penetrating peptides (CPPs) have shown enhanced uptake and delivery of siRNA in the cells [21,22,23]. Tat or polyarginine-containing peptides were among key CPPs that have been used extensively in cargo delivery, including siRNA delivery, due to the presence of multiple positively charged guanidinium groups [24,25,26]. We have reported an alternate combination of histidine and arginine amino acid (HR)_4_ as a molecular transporter of small molecule and phosphopeptide [27]. Recent studies demonstrated the use of histidine or arginine-containing CPPs to transport siRNA [28,29]. Furthermore, the combination of arginine with histidine residues was reported to modulate the cytotoxicity of the siRNA delivery system [30,31].

Fatty acylation has been reported to increase cellular uptake of CPPs with low cytotoxicity and enhanced siRNA transfection efficiency [27,29,32]. Alshamsan et al. reported that hydrophobically modified PEI by oleic acid and stearic acid increased siRNA condensation, protection of siRNA from FBS degradation, and cellular uptake of siRNA as compared to the parent PEI [33]. It has also been reported that the *N*-terminal stearylation of arginine-rich peptides increased the transfection efficacy because hydrophobic moiety contributes to the absorption of the complex on the membranes [34]. Similarly, Sharma et al. demonstrated that the oleyl-conjugated CGKRK peptide showed the highest efficiency in siRNA uptake and silencing of kinesin spindle protein (KSP) compared to other saturated fatty acid conjugated peptides [35]. Therefore, herein, we sought to extend oleic acid conjugation to histidine–arginine-based peptides to develop an efficient and safer siRNA delivery system. We hypothesized that conjugation of oleic acid to histidine and arginine residues (HR)_4_ would result in an amphiphilic molecule with optimal cationic charge and hydrophobic bulk required for the efficient siRNA delivery. Net cationic charge/hydrophobic bulk ratio dictates the selective membrane permeabilizing ability of the CPPs. The cationic charge will be increased by the addition of arginine (R) residue between hydrophobic oleyl and cationic (HR)_4_ residues. Moreover, increasing the hydrophobicity of the siRNA delivery system using unsaturated oleic acid has been shown to enhance cellular uptake [36]. Therefore, to identify the optimum charge/hydrophobic bulk ratio in our unique amphiphilic delivery system, a series of fatty acylated peptides was designed by incorporating an increasing number of R residues, i.e., C_18*_-(R)_n_-(HR)_4_, (where C_18*_ represents oleyl group and n = 0, 1, 2, 3, 4 or 5). 

To the best of our knowledge, limited studies have reported the use of HR peptide-based carriers for siRNA delivery in triple-negative breast cancer cells (TNBC) [31]. However, the role of varying R residues in improving the cellular uptake of siRNA and modulation of the cytotoxicity of the peptide has not yet been performed. In this study, we combined hydrophobicity enhancing motif (oleic acid), cationic peptide (HR)_4_, and varying R residues to develop an efficient siRNA delivery system for TNBC cell lines and evaluate the silencing of model protein signal transducer and activator of transcription -3 (STAT-3).

## 2. Materials and Methods

The protected building block amino acids (arginine and histidine) and arginine preloaded on 2-chlorotrityl resin were purchased from Chem Impex Inc. (Bensenville, IL, USA). 2-(1H-benzotriazole-1-yl)-1,1,3,3-tetramethyluronium hexafluorophosphate (HBTU) was purchased from Acrotein ChemBio Inc. (Hoover, AL, USA). *N, N*-diisopropylethylamine (DIPEA) and all other reagents and chemicals for peptide synthesis were obtained from Millipore Sigma (Milwaukee, WI, USA). Silencer^®^ Negative Control #1 siRNA, Catalog #: AM4635-AM4636 was purchased from Ambian Inc. (Austin, TX, USA). Negative control siRNA, also called scrambled siRNA, was the siRNA with 24 nucleotide sequences and does not target any gene product. The negative control siRNA was used to make siRNA-Peptide conjugate complexes in order to perform binding affinity, serum stability, cytotoxicity assay, and dynamic light scattering (DLS) experiments. Alexa Fluor 488 labeled nonsilencing control siRNA with the sequence 5′-AAT TCT CCG AAC GTG TCA CGT-3′ was purchased from QIAGEN Sciences (Germantown, MD, USA). Alexa-488 labeled nonsilencing control siRNA was used to perform cellular uptake studies using flow cytometry and confocal microscopy. Hs_STAT3_7 FlexiTube siRNA was purchased from QIAGEN LLC (Germantown, Maryland, USA). The target sequence was 50-CAGCCTCTCTGCAGAATTCAA-30, the sense strand was 50-GCUUCUCUGCAGAAUUCAATT-30 and the antisense strand was 50-UUGAAUUCUGCAGAGAGGCTG-30. STAT-3 (124H6) Mouse mAb #9139 and GAPDH (D4C6R) Mouse mAb #97166 were purchased from Cell Signaling Technology (CST), (Danvers, MA, USA), for the Western blotting experiment. Horseradish peroxidase (HRP)-linked secondary antibody was purchased from Abcam Inc. (Waltham, MA, USA). Mini-PROTEAN TGX stain-free precast gels and Trans-Blot Turbo Mini 0.2 m PVDF transfer packs #1704156 were purchased from Bio-Rad Inc. (Hercules, CA, USA). All other reagents used for Western blotting experiments were purchased from Bio-Rad Inc (Hercules, CA, USA).

### 2.1. Synthesis and Purification of Oleyl Conjugated Peptides

**Scheme 1:**
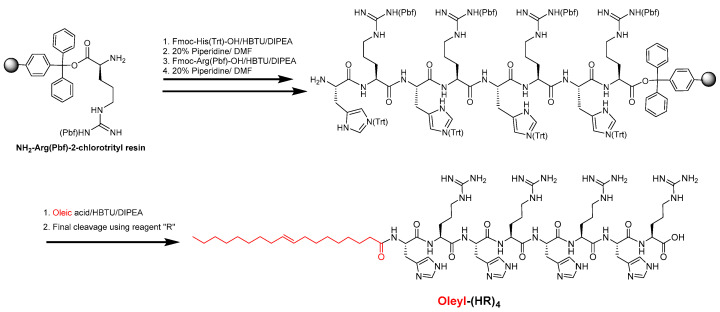
Synthesis of Oleyl-(HR)_4_ peptide.

The oleic acid conjugated peptides were synthesized using Fmoc/tBu solid-phase synthesis, as depicted by the representative example in Scheme 1 using previously published reports [27,35]. Briefly, the preloaded NH_2_-Arg(Pbf)-2-chlorotrityl resin as solid support (0.30 mmol scale) was used to assemble peptide containing an alternate arginine and histidine residue as per the sequence of peptides, followed by *N*-terminal acylation with oleic acid using 2-(1h-benzotriazole-1-yl)-1,1,3,3-tetramethyluronium hexafluorophosphate/N, N-diisopropylethylamine (HBTU/DIPEA). Finally, all the peptide conjugates were cleaved using a freshly prepared cleavage cocktail reagent R (trifluoroacetic acid (TFA): thioanisole: anisole: 1,2-ethanediol (EDT); 92:5:2:3; *v/v/v/v*) for 4 h, and peptides were precipitated using cold diethyl ether, centrifuged and purified using reverse-phase high-performance liquid chromatography (RP-HPLC) (Shimadzu, LC-20AP Prominence) with a gradient system of water (H_2_O), acetonitrile (CH_3_CN) in 0.1%TFA (*v/v*) (5−90%, 50 min) on a reversed-phase preparative column (00G-4436-P0-AX, Gemini Prep C18, 10 μm particle size). The fractions were collected and analyzed using matrix-assisted laser desorption/ionization time-of-flight (MALDI-TOF) mass spectrometer (GT 0264, Bruker, Inc. Billerica, MA, USA). Fractions showing a mass of expected compounds were pooled and lyophilized to obtain a solid powder of peptides. Analytical HPLC was performed to further confirm the purity of the compounds ≥ 95%, which were used in the biological assays. The chemical structures, MALDI mass spectra and purity data of the compounds are shown in the Supplementary Information. Table 1 represents the sequences of the peptide conjugates and their MALDI mass characterization data.

### 2.2. Complex Formation of Oleyl Conjugated Peptides and siRNA

The complex of oleyl conjugated peptides and siRNA was formed through physical mixing. Appropriate volumes of oleyl conjugated peptides and siRNA solution were mixed in Hank’s balanced salt solution (HBSS) buffer to obtain the complexes. The oleyl conjugated peptide-siRNA complexes were formed due to ionic interaction between positively charged arginine residues of the conjugates and the negatively charged phosphate groups of siRNAs. The final concentration of siRNA in these complexes was kept constant while the concentration of the conjugates gradually increased to obtain different N/P ratios. The N to P ratio is commonly used where N represents the number of moles of ionizable nitrogen in the delivery agent. In contrast, P refers to the number of moles of phosphates present in siRNA. The ratio is calculated using the following formula.

$$\frac{N}{P}=\frac{\mathrm{number}\;\mathrm{of}\;\mathrm{moles}\;\mathrm{pf}\;\mathrm{peptidex}\;\mathrm{number}\;\mathrm{of}\;\mathrm{ionized}\;\mathrm{nitrogen}\;\mathrm{atoms}}{\left(\mathrm{number}\;\mathrm{of}\;\mathrm{moles}\;\mathrm{of}\;\mathrm{siRA}\;\times 48\right)}\;$$

### 2.3. Cell Culture

Triple-negative breast cancer cell line MDA-MB-231 (ATCC^®^ No. HTB-26), breast adenocarcinoma MCF-7 cells (ATCC^®^ No. HTB-22) and normal breast cells MCF-10A (ATCC^®^ No. CRL10317) were purchased from American Type Culture Collection (ATCC) (Manassas, VA, USA). DMEM media, supplemented with fetal bovine serum (FBS, 10%) and penicillin-streptomycin solution (10,000 units of penicillin and 10 mg of streptomycin in 0.9% NaCl, 1%), were used for the proliferation of MDA-MB-231 and MCF-7 cells. MEGM Kit (catalog # CC-3150) was purchased from Lonza Group Ltd. (Muenchensteinerstrasse 38, CH-4002 Basel, Switzerland) for the proliferation of the MCF-10A cell line. The kit consists of MEBM Basal Medium (CC-3151) and MEGM Supplement pack (CC-4136) containing an orange cap vial with bovine pituitary extract (BPE), green cap vial with human epidermal growth factor (hEGF), Lilac cap vial with insulin, natural cap vial with hydrocortisone, and red cap vial with gentamicin sulfate-amphotericin (GA-1000). All the cell lines used in this study were incubated in a humidified atmosphere of 5% CO_2_ and 95% air at 37 °C. The cells were handled under sterile conditions in Herasafe™ 2030i Class 2 A2 Biological Safety Cabinets purchased from Thermo Scientific™ (Hanover Park, IL, USA). All supplies for the cell culture experiments were obtained from Thermo Fisher Scientific.

### 2.4. In Vitro Cytotoxicity Assay

The in vitro cytotoxicity of the synthesized oleyl-conjugated peptides complexed with scrambled siRNA at different N/P ratios ranging from 10 to 100 was determined in the MDA-MB-231, MCF-7, and MCF-10A cell lines. The peptide-siRNA complexes were made according to the protocol described in Section 2.2. A total of 10,000 cells per 0.1 mL were seeded in each well in a 96-well plate using a multichannel pipette and were allowed to adhere to the bottom of the plate for 24 h in the incubator. After 24 h, the cells were inspected for their health and confluency. Different concentrations of the freshly prepared peptide-siRNA complexes were added to each well in triplicate and incubated for 48 h at 37 °C in a humidified atmosphere of 5% CO_2_. After 48 h, 20 μL of the 3-(4,5-dimethylthiazol-2-yl)-5-(3-carboxymethoxyphenyl)-2-(4-sulfophenyl)-2H-tetrazolium (MTS) reagent was added to each well using a multichannel pipette. The 96-well plates were centrifuged at 1000 rpm (RCF =96× *g*) for 1 min to ensure the settling of the MTS reagent, and after that, the plates were incubated for an additional 3 h. SpectraMax M2 microplate spectrophotometer was used to determine the absorbance at 490 nm. The percentage cell viability was calculated using the following formula

$$\%\;\mathrm{cell}\;\mathrm{viability}=\left(\frac{\mathrm{Average}\;\mathrm{OD}\;\mathrm{values}\;\mathrm{of}\;\mathrm{samples}\;\mathrm{treated}\;\mathrm{with}\;\mathrm{the}\;\mathrm{compound}}{\mathrm{Average}\;\mathrm{OD}\;\mathrm{values}\;\mathrm{of}\;\mathrm{no}\;\mathrm{treatment}}\right)\times 100\;$$

### 2.5. Dynamic Light Scattering

The peptide-siRNA complexes were characterized using the dynamic light scattering (DLS) technique. Malvern Nano ZS Zetasizer (Westborough, MA, USA) was used to determine the hydrodynamic diameter and surface charge of the complexes at 25 °C. The zeta potential of the complexes was determined at 40 V using disposable folded capillary cells (DTS1070). The calibration of the instrument was performed by transfer standard DTS 1235. The disposable cuvettes were used for determining the hydrodynamic diameter. The N/P ratio 40 was selected for performing the dynamic light scattering experiment. At this N/P ratio, the final concentration of siRNA in the complex was 50 nM whereas the concentration of oleyl-(HR)_4_ was 24 µM, oleyl-R_1_-(HR)_4_ was 19.20 µM, oleyl-R_2_-(HR)_4_ was 16 µM, oleyl-R_3_-(HR)_4_ was 13.71 µM, oleyl-R_4_-(HR)_4_ was 12 µM, and oleyl-R_5_-(HR)_4_ was 10.67 µM. The final volume of the complex used for performing zeta potential was 750 µL. The measurements were performed in triplicate, where each measurement consisted of 20 runs. The zeta potential values were calculated using the Smoluchowski model. All the results passed the quality standard of the instrument.

Particle size was also determined at N/P 40 with the same concentrations of siRNA and oleyl conjugated peptides as described above. The experiments were performed in triplicate on each sample with an automatic attenuator setting. The results were checked with the quality standards of the instruments and were represented as mean ± SD of three independent experiments.

### 2.6. Cellular Internalization of siRNA

The cellular uptake of siRNA complexed with oleyl conjugated peptides was evaluated using flow cytometry (BD-FACSVerse; BD Biosciences, San Jose, CA, USA) in live cells. Alexa fluor 488-labeled scrambled siRNA was used for making complexes with oleyl conjugated peptides. The peptide-siRNA complexes were made at N/P 40; the final concentration of siRNA in each complex was 50 nM. The study was performed on MDA-MB-231 according to the protocol described by Mandal et al. [37]. Briefly, approximately 500,000 cells were seeded in each well of the six-well plates and were allowed to settle for 24 h. The cells were treated with the conjugate-siRNA complexes (N/P 40) and were incubated in a humidified atmosphere of 5% CO_2_ and 95% air at 37 °C for 24 h. After 24 h of treatment, the cells were washed three times with HBBS and were trypsinized. The suspended cells were centrifuged for 5 min at 1000 rpm (96× *g*) at 4 °C, and the cells pellet was washed with HBSS three times to remove any traces of the medium. Finally, the cells were resuspended in PBS and analyzed by flow cytometer using Alexa-Fluor-488 channel to quantify the mean fluorescence intensity. The mean fluorescent intensity in the treated cells was calculated for each sample based on the calibration of the signal gated with nontreated cells, serving as a negative control. Alexa-488 labeled scrambled siRNA served as a second negative control, and lipofectamine-siRNA treated cells were used as a positive control.

### 2.7. Cellular Uptake Study Using Confocal Microscopy

Confocal microscopy was used to corroborate the data obtained from flow cytometry. There have been reports for using fixed cells [35,38,39,40] or live cells [41] for imaging the cellular uptake of siRNA. We adopted the protocol of Aliabadi et al. [38] to perform the confocal microscopy. Briefly, approximately 400,000 MDA-MB-231 cells were seeded on coverslips in 6-well plates and were allowed to settle for 24 h. After 24 h, the cells were visualized under an ordinary microscope for their health and confluency. Peptide-siRNA complexes at N/P ratio 40 were made containing a final concentration of 50 nM of siRNA, and the cells were treated with these complexes. Nontreated cells served as negative control while lipofectamine-siRNA complexes were used as a positive control. After 24 h of treatment, the cells grown on coverslips were washed 3 times with HBSS and fixed with 3.7% formaldehyde solutions solution for 30 min at room temperature. The cell membranes of the cells were stained with Texas Red (TR) Phalloidin (Invitrogen) (1:250 in HBSS), while the nuclei of the cells were stained with 4′,6-diamidino-2-phenylindole (DAPI). Nikon A1R confocal microscope system was used to image the fixed cells at 60X objective and different filters for Alexa-488 (FITC), DAPI, and TR.

### 2.8. Gel Shifting Assay

Gel electrophoresis was performed to determine the binding affinity of the oleyl conjugated peptides with scrambled siRNA. The peptides mentioned in Table 1 were mixed with siRNA at N/P ratios ranging from 0 to 40, where the final concentration of siRNA in the complexes was 50 nM. In contrast, the peptide’s concentration increased with an increase in the N/P ratio. The complexes were made in HBSS buffer and were placed at room temperature for adequate complexation of the siRNA and the peptides. N/P ratio 0 served as a negative control in which no peptide was present. One percent agarose gel was freshly prepared and stained with 0.5 μg/mL ethidium bromide. A total of 5 µL of gel loading dye, purple (6X) (catalogue #B7024, New England Biolabs) (Ipswich, MA, USA), was added to each complex before loading into the wells. The gel electrophoresis was carried out at 70 V, 400 mA for 20 min in TAE buffer (2 M Tris base, 1 M glacial acetic acid, 0.5 M sodium EDTA, pH 8.3). The ChemiDoc XRS+ system, based on CCD high-resolution (Bio-Rad Imager), was used to visualize the gel, and the intensity of the bands (representing unbound siRNA) was quantified using Image Lab™ software. The experiment was performed in triplicate; the representative images of the gels are represented in the results sections of the manuscript. Percent binding (%Binding) was calculated as 100% × [(control siRNA − free siRNA)/control siRNA].

### 2.9. Protection of siRNA against Enzymatic Degradation

Oleyl conjugated peptides were examined for their ability to protect the siRNA from the harsh biological environment consisting of nucleases that rapidly degrade siRNA. For this purpose, the peptide-siRNA complexes were prepared with N/P ratios ranging from 0 to 40, with a final siRNA concentration in these complexes as 100 nM. N/P ratio 0 served as a positive control, representing 100% intact siRNA. HBSS was used as a negative control. Each of the peptide-siRNA complexes of different N/P ratios was exposed to 25% (*v/v*) FBS solution in HBSS, and the mixture was incubated at 37 °C for 24 h. After 24 h, the heparin competition assay was used to dissociate the peptide-siRNA complex and determine the amount of intact siRNA after exposure to FBS. For this purpose, 5 µL of a 2:3 mixture (*v/v*) of heparin (5% solution in normal saline) and ethylenediaminetetraacetic acid (0.5 mM) was added to each complex and left for 10 min. A total of 5 µL of gel loading dye, purple (6X), was added to each mixture, and the samples were analyzed using 1% ethidium bromide gel electrophoresis run at 70 V, 400 mA for 20 min. The gels were visualized under UV illumination, and the intensity of the bands was determined using Image Lab™ software. Three independent experiments were performed for each oleyl conjugated peptide. The representative gel images are shown in the results section of the manuscript.

### 2.10. Protein Silencing Effect of siRNA (Western Blot)

STAT-3 is an essential protein overexpressed in several cancer types, including breast cancer [42]. The silencing effect of the STAT-3 siRNA delivered via oleyl conjugated peptides was determined by checking the expression of STAT-3 in peptide-siRNA complex treated cells. Expression of STAT-3 in nontreated cells served as a negative control, whereas the commercially used transfection agent lipofectamine (20 µg/mL) complexed with STAT-3 siRNA (50 nM) served as the positive control. STAT-3 expression was evaluated by the Western blotting experiment. Approximately 500,000 MDA-MB-231 cells were seeded in T-25 flasks and incubated at 37 °C under standard growth conditions. After 24 h, the cells were treated with peptide-siRNA complexes at N/P 40 for 48 h. The final siRNA concentration at this N/P was 50 nM. After 48 h of treatment, MDA-MB-231 cell lysates were prepared according to the standard protocol using RIPA buffer; the cell lysates were then incubated on ice for 1 h, during which the tubes were sonicated for 3 min after every 10 min. Following sonication for 1 h to ensure the complete lysis of cells, the tubes were centrifuged for 15 min at 12,000 rpm (RCF = 13,870× *g*) at 4 °C. The supernatant from the centrifuged tubes was transferred to a fresh set of pre-cooled microtubes, and the total protein concentration was determined using BSA assay. Briefly, 200 μL of working reagent (50:1 A:B) was added to 25 μL of standard and unknown samples in triplicate into a 96-well plate, and the sample was mixed on a plate shaker for 30 s. The plate was then incubated at 37 °C and 5% CO_2_ for 30 min. After half an hour of incubation, the absorbance was measured at 562 nm using a Spectra MAX M5 microplate reader. The protein in each treatment sample was quantified using the BSA standard curve. A total of 15 µg of the protein was loaded per well in a 10% Mini-PROTEAN^®^ TGX Stain-Free. Protein gel electrophoresis was performed in buffer (0.192 M glycine, 25 mM Tris, 0.1% SDS) for 30 min at 200 V. The gel was then transferred to Mini PVDF membrane (Catalog no. 1704156) using Trans-Blot^®^ Turbo (Bio-Rad, Hercules, CA, USA). The membrane was blocked in BSA 5% for 3 h and then incubated at 4 °C overnight with the primary antibody (1:1000 in TBS-T). The membrane was then washed with TBS-T six times (5 min each time) and was subsequently incubated with the secondary HRP-linked antibody (1:1000 in TBS-T) for 1 h, followed by the washing steps. The detection of the bands was done by ECL Detect Kit using a ChemiDoc imager (Bio-Rad, Hercules, CA, USA). The bands were quantified using Image Lab™ software of ChemiDoc imager.

### 2.11. Statistical Analysis

Data obtained from the above-described experiments are shown as mean ± SD of three independent experiments unless stated otherwise. Ordinary one-way analyses of variance (ANOVA) with multiple comparisons using Bartlett’s test were conducted to analyze flow cytometry and Western blotting data. Paired Student’s *t*-test was performed for comparison among two groups. *p*-value < 0.05 was considered to be statistically significant.

## 3. Results and Discussion

### 3.1. Synthesis and Characterization of the Oleyl-Conjugated Histidine-Arginine-Containing Peptide

CPPs are 5−40 amino-acid-long peptides known for their ability to internalize the cargo into the cell, and they have been used for siRNA delivery [23,43]. Positively charged amino acids, such as arginine and lysine, are required for complex formation with siRNA [32]. It has been suggested that intracellular delivery could be optimized by adjusting the number of arginines in the peptide [44]. For instance, Tanaka et al. reported that stearoyl -CH_2_R_4_H_2_C peptide conjugate could be an effective siRNA carrier to exercise silencing effect in vitro and in vivo [30]. Moreover, Dutta et al. reported that arginine-based CPPs with side-chain modification of two stearyl moieties exhibited significantly enhanced cellular internalization of siRNA even with only six L-arginine residues. This study infers that lipopeptides having Arg-Sar-Arg motif and two stearyl moieties exhibit higher siRNA internalization efficacy than stearylated nona-arginine and can be exploited as a promising drug delivery vehicle for intracellular delivery of biomacromolecules [45].

Several studies [7,16,17,23,27,45] have used histidine and arginine-based CPPs to improve their effectiveness in delivering the siRNA and increasing the transfection efficiency. In oligoarginine-based siRNA transporters, adjacent Arg−Arg repulsion between two consecutive arginine residues and proteolytic instability of transporters limit the stability of peptide−cargo complexes [45,46]. The surface charge of nanocomplexes can affect cellular uptake, cytotoxicity, tumor penetration, and therapeutic efficacy [47,48]. Additionally, the internalization of peptide–cargo complexes by endocytic pathways and endosomal entrapment prevents cytosolic delivery of siRNA [49,50]. Polyethyleneimine-containing polymer or delivery vehicle having histidine residues exhibit proton sponge effect and facilitate the release of encapsulated cargo to the cytosol [51]. MacLachlan and coworkers have demonstrated that unsaturation in cationic lipids can affect fusogenicity and cellular uptake of delivering nucleic-acid-based therapeutic molecules [52]. Moreover, the substitution by histidine in oligoarginine peptide has proven to improve the safety of the peptide allowing the optimization of the peptide structure mainly by changing its binding energy and the 3D-hydrophobic moment [53]. The designed library of oleyl conjugated HR containing CPPs proved to be comparatively less toxic to stearyl nona-arginine CPP [45]. Another interesting finding that led us to formulate and design the library of the peptides mentioned above is that adding histidine to an oligoarginine CPP decreases the toxicity and improves the effectiveness of the peptide [53]. Few studies have been conducted lately to discover the role of arginine residues in lipid-based siRNA transfection agent. Recently, Konate et al. conducted a comprehensive study in which they designed novel amphipathic cell-penetrating peptides, called WRAP, for siRNA transfection [54]. The current study is designed on the same lines to discover the lipid modified amphiphilic CPPs for efficient siRNA delivery.

Peptides in Table 1 were synthesized using Fmoc/tBu solid-phase peptide chemistry as depicted in Scheme 1 with the representative synthesis of oleyl-(HR)_4_ peptide. All the peptides were characterized by MALDI-TOF mass spectroscopy (Table 1) and purified using RP-HPLC. The analytical HPLC showed a purity of ≥95%. The spectra of mass data, as well as the purity data of the synthesized conjugates, are provided in the Supplementary Information (Figures S1–S12).

### 3.2. Cytotoxicity

The cytotoxicity profile of the peptide-siRNA complexes was determined to find the safe N/P ratio (quantity of the peptide/siRNA) for cellular uptake and gene silencing studies. Therefore, two breast cancer cell lines, MDA-MB-231 and MCF-7, and one normal breast epithelial cell line (MCF-10A) were used. The peptide-siRNA complexes were formed at different N/P ratios ranging from 10 to 100. The siRNA concentration in each complex was constant and was chosen as 1000 nM to test the peptides at higher concentrations. The concentration of the peptides increased with increasing N/P. Moreover, the peptide concentration depended upon the amount of ionizable nitrogen in the peptides; with more ionizable nitrogen, the peptide concentration decreased. For instance, the relationship of N/P to the concentration of peptides at N/P 40 is presented below in Table 2. 

**Figure 1:**
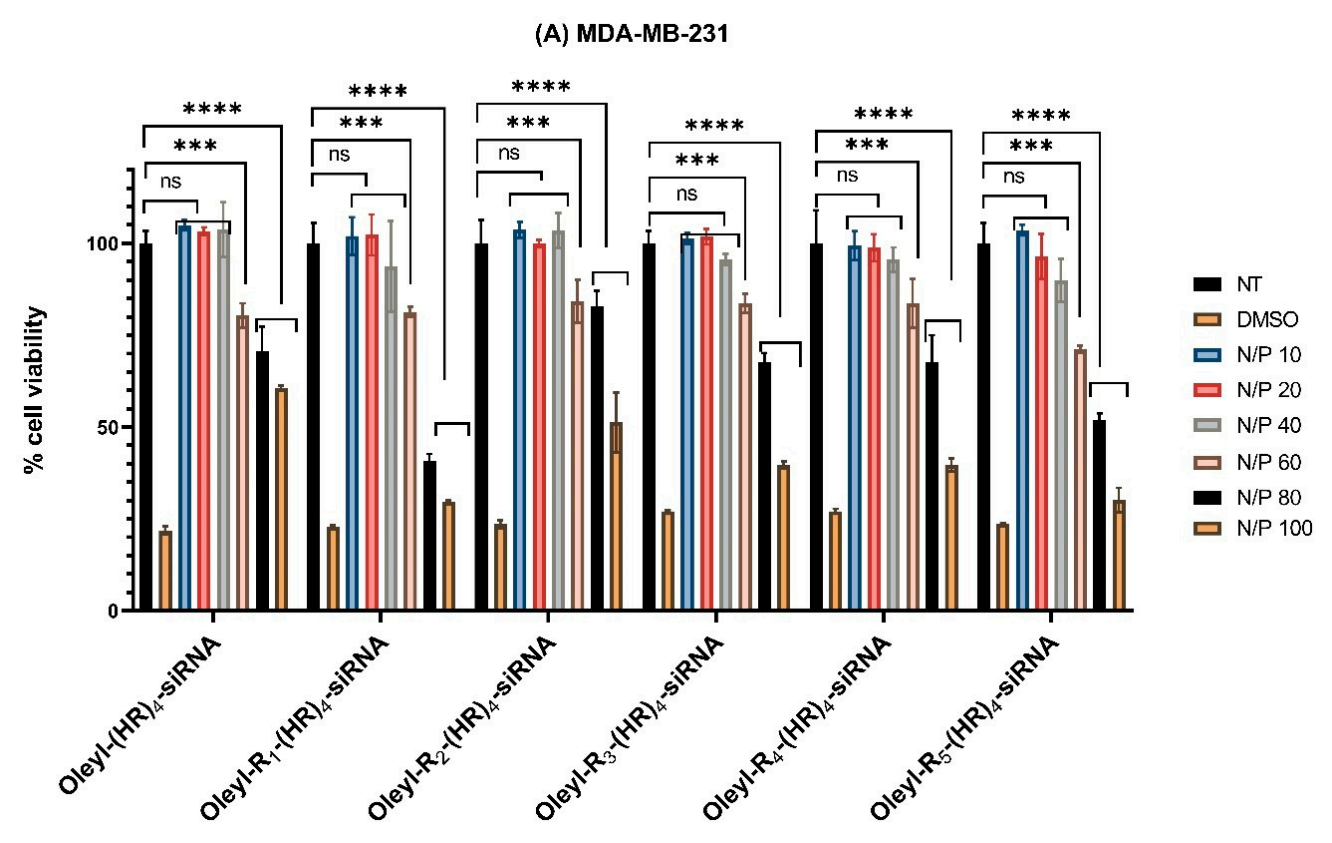

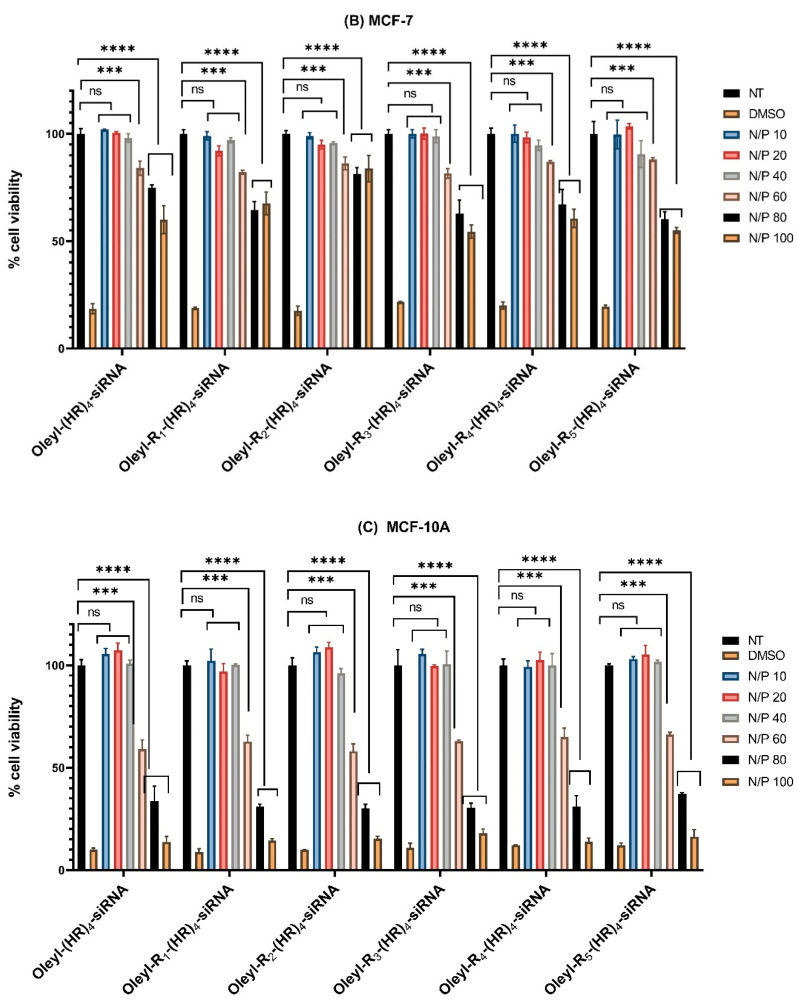
(**A**) Cytotoxicity of peptide/siRNA complex in MDA-MB-231 cells (**B**) Cytotoxicity of peptide/siRNA complex in MCF-7 cells (**C**) Cytotoxicity of peptide/siRNA complex in MCF-10A cells. Incubation time in each assay was 48 h. Graphs represent mean normalized values (based on No Treatment or NT group; *n* = 3). ns means nonsignificant. (if *p* < 0.001 then ***, if *p* < 0.0001 then ****). Error bars indicate standard deviation. Thirty-five percent *v*/*v* DMSO served as a positive control.

The cytotoxicity data are presented in Figure 1A–C below. The data shows that all the peptide-siRNA complexes showed nonsignificant toxicity compared to nontreated cells (*p* < 0.05) in all the three cell lines at N/P 40. However, with increase N/P ratio and subsequently the concentration of the peptides, the complexes showed significant toxicity. Therefore, N/P 40 was selected as a safe ratio of peptide siRNA complexes for further assays to ensure the cells were alive during the experimental conditions (see below, Section 3.4 and Section 3.8).

### 3.3. Characterization of Peptide-siRNA Complexes Using Dynamic Light Scattering

#### 3.3.1. Zeta Potential

**Figure 2:**
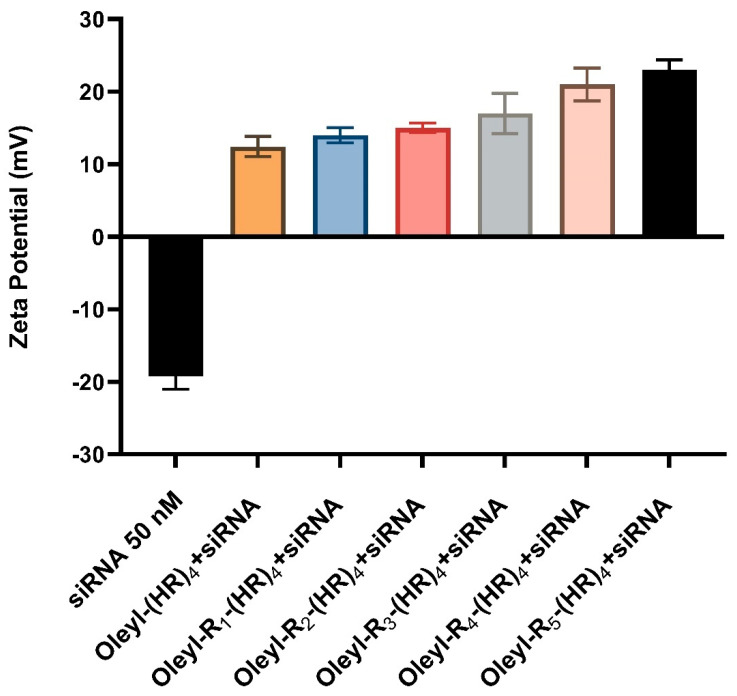
Zeta potential values of peptide-siRNA complexes, where siRNA concentration in each complex in 50 nM and the concentration of the peptide varied according to their structure at N/P 40. Error bars indicate the standard deviation. Each experiment was performed independently in triplicate.

Zeta potential is an important technique in determining surface charge on the complexes and evaluating the complexes’ degree of aggregation in the solution. The zeta potential was determined using Malvern Nano ZS Zetasizer for each peptide-siRNA complex, as shown in Figure 2 below. siRNA concentration in each of the complexes was 50 nM, whereas the peptide concentration varied with the amount of ionizable nitrogen. N/P ratio for each complex was chosen as 40. The data indicates the peptides-siRNA complexes show an overall positive zeta potential ranging from +10 mV to slightly over +20 mV. With an increase in arginine residues, the zeta potential value increased. The surface charge of the complexes could affect their interaction with the cell membrane and, therefore, their cellular internalization. The positive zeta potential values indicate the stability of the complexes [55], and possibly favorable interaction with cell membranes, which are important attributes for a successful siRNA delivery system. 

#### 3.3.2. Particle Size

The size of the siRNA-oleyl conjugated complexes can affect the cellular internalization efficiency of the siRNA. We determined the hydrodynamic diameter of the siRNA-oleyl conjugated peptides using the dynamic light scattering method. The hydrodynamic diameter was evaluated at N/P 40. The results are presented in Table 3. The particle size of the studies’ complexes ranged from ~115 nm to ~120 nm. This small change in the diameter of the complexes may be due to a single arginine residue difference between the conjugated peptides. The data shows that the oleyl conjugated-siRNA complexes self-assembled in which the oleyl side chain may have contributed significantly due to hydrophobic interaction.

The polydispersity index (PDI) gives the physical stability of nanosuspensions and should be as low as possible for the long-time stability of nanosuspensions. A PDI value of 0.1 to 0.25 shows a fairly narrow size distribution, and PDI value more than 0.5 indicates a very broad distribution of the polydispersity index [56]. The PDI values of ~0.2 indicate less aggregation of the complexes (Table 3). Previously, Tanaka et al. reported the particle size of ~103 nm for stearyl conjugated-CH_2_R_4_H_2_C/siRNA complex at N/P 20 [30]. Moreover, Biswas et al. studied several linear and cyclic stearyl and linoleyl conjugated peptides for siRNA delivery and found the size in a range of 100 to 120 nm [31]. Based on the previously reported data and the results from this study, it is plausible to speculate that fatty-acyl conjugated amphiphilic peptides form complexes with siRNA in a nano-range that contributes to their ability to internalize siRNA inside the cells. 

### 3.4. Cellular Internalization of Alexa Fluor Labeled siRNA Using Flow Cytometry

Cellular uptake of siRNA is a crucial step required for its ability to bind to siRNA-induced silencing complex (RISC) with subsequent degradation of mRNA of the desired gene. Once inside the cell, the siRNA that presumably internalizes via endocytosis along with the carrier, the endosome must release the siRNA to make it able to bind to RISC [4]. Cellular uptake study of the oleyl-conjugated-siRNA complexes at N/P 40 was performed on MDA-MB-231 cells using flow cytometry as described earlier in Section 2.6. MDA-MB-231 cells are relatively more resistant to transfection than other breast cancer cell types [28,57].

**Figure 3:**
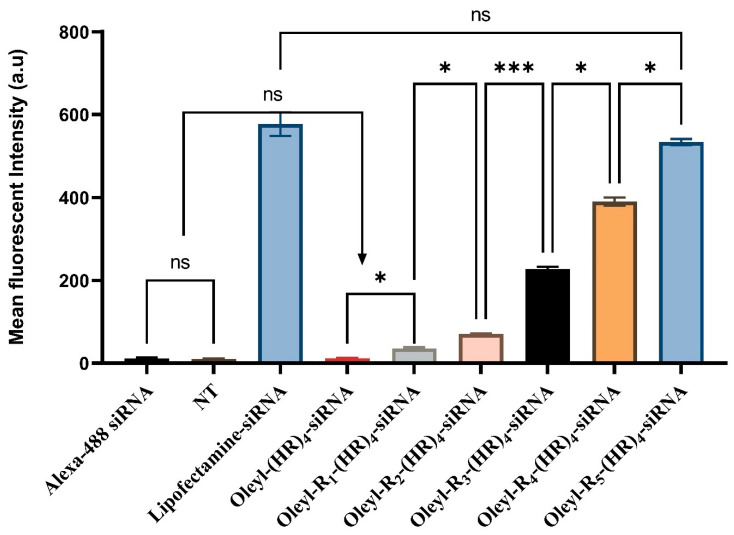
Cellular internalization of oleyl conjugated-siRNA complexes in MDA-MB-231. Bars indicate the mean fluorescent intensity of Alexa fluor-488 labeled siRNA. The error bars indicate ± SD (*n* = 3), ns means nonsignificant, * indicates *p*-value < 0.05, *** indicate *p*-value < 0.001.

Alexa Flour-488 labeled siRNA is commonly used to determine siRNA’s cellular internalization using fluorescence-activated cell sorting (FACS) flow cytometry (Figure 3). Nontreated cells (NT) and Alexa-488 siRNA treated cells were used as a negative control, lipofectamine-siRNA was used as positive control. The results suggest that there was nonsignificant difference in fluorescent intensity between nontreated cells and Alexa-488 treated cells (*p* = 0.639). Although, there was a slight increase in fluorescent intensity between oleyl-(HR)_4_ and oleyl-R_1_-(HR)_4_ (*p* = 0.0447). However, there was nonsignificant difference in intensity when compared with Alexa-488 treated cells (*p* = 0.9988 for oleyl-(HR)_4,_ and *p* = 0.0504 for oleyl-R_1_-(HR)_4_). There was a significant increase in mean fluorescent intensity and the siRNA uptake by the cells with successive increase in arginine residues, with the maximum cellular uptake observed by oleyl-R_5_-(HR)_4._ Moreover, a nonsignificant difference in cellular uptake of lipofectamine and oleyl-R_5_-(HR)_4_ was observed, which can be corroborated with the comparable silencing efficiency of both compounds. Based on the cellular uptake results, we did not perform further assays on oleyl-(HR)_4_ and oleyl-R_1_-(HR)_4_.

### 3.5. Cellular Internalization Using Confocal Microscopy

**Figure 4:**
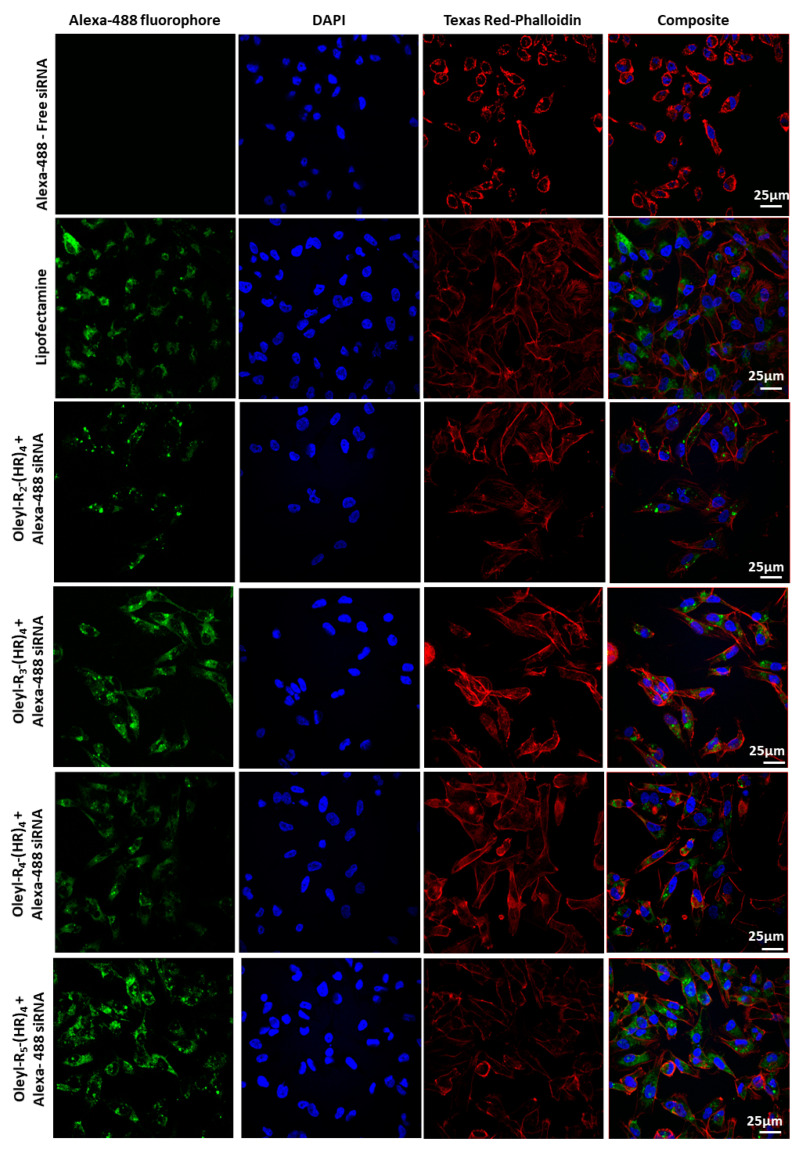
Confocal microscopy images of MDA-MB-231 cells treated with oleyl-conjugated (HR)_4_ peptides.

Confocal microscopy is an essential technique for qualitatively visualizing the cellular internalization of fluorophore-conjugated siRNA. As mentioned earlier in Section 2.7, there have been reports for using fixed cells [35,38,39,40] or live cells [41] for imaging the cellular uptake of siRNA. We performed microscopy in the fixed cells as reported by Aliabadi et al. [38]. After treatment, rigorous washing of cells was performed to ensure complete elimination of any peptide:siRNA complexes on the cell surface before fixing the cells and subsequent staining to visualize the cellular uptake of siRNA in the cells. Free siRNA (with no peptide) was used as a negative control, whereas the commercial transfection reagent Lipofectamine 2000 was used as a positive control. The confocal microscopy results (Figure 4) corroborate the quantitative data obtained by flow cytometry (Figure 3). Oleyl-R_4_-(HR)_4_ and Oleyl-R_5_-(HR)_4_ showed a comparable green fluorescence (Alex-488 fluorophore), representing that these peptides show promise in delivering siRNA efficiently into the MDA-MB-231 cells.

### 3.6. Binding Affinity of the Peptides with siRNA Using Gel Electrophoresis

**Figure 5:**
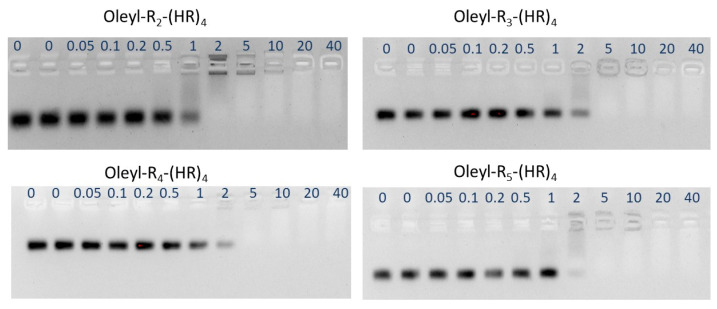
Representative images of gel retardation assay for evaluating binding affinity of the oleyl conjugated peptides with the siRNA.

Gel retardation assay was used for determining the amount of peptide that retards siRNA migration. Several N/P ratios ranging from 0 to 40 were used. All the peptide conjugates showed an adequate retardation of siRNA at N/P ratio ≥ 2 (Figure 5). siRNA concentration in each complex was 50 nM

**Figure 6:**
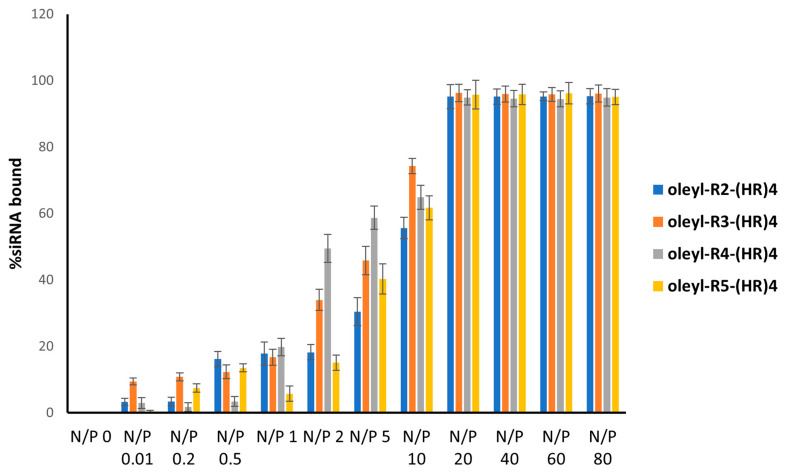
Gel retardation assay of oleyl-conjugated peptides/siRNA complexes at N/P ratios ranging from 0 to 80. The bars indicate the mean intensity of the bands, representing percent siRNA bound to the peptides, quantified by Image Lab software. Error bars show the standard deviation (*n* = 3).

The quantification of the bands was done by the Image Lab software that represented the percent bound siRNA to the peptides. The decrease in band intensity represents that the siRNA is bound with the oleyl conjugated peptide, and hence its movement was retarded in the gel. At N/P 0, indicating only the siRNA, all the siRNA showed up as a thick band; however, with an increase in N/P ratio (increase in peptide concentrations in the complexes), percent bound siRNA increased, showing ~100% bound siRNA at N/P 20 and above (Figure 6). The binding affinity of the oleyl conjugated peptides is probably due to the presence of arginine residues in the peptides that made complexes with siRNA through ionic interactions with negatively charged phosphates in the siRNA.

### 3.7. Serum Stability of Peptide-siRNA Complexes

**Figure 7:**
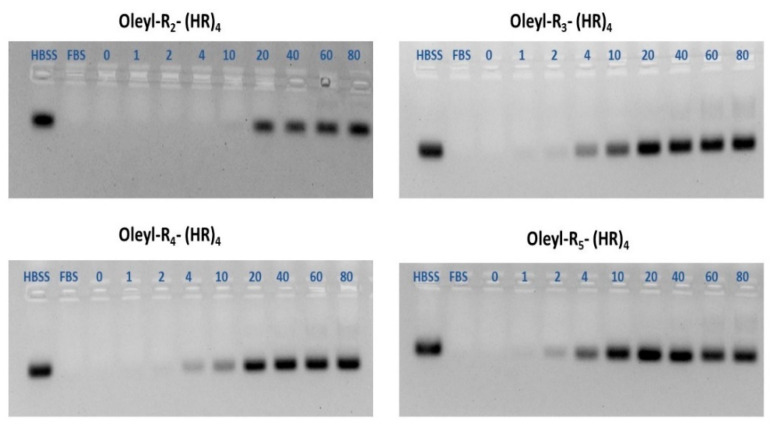
Representative images of gel retardation assay for evaluating the ability of the peptides to protect siRNA from 25% FBS after 24 h treatment. The gel was run after dissociating the peptides/siRNA complexes using a heparin competition assay.

**Figure 8:**
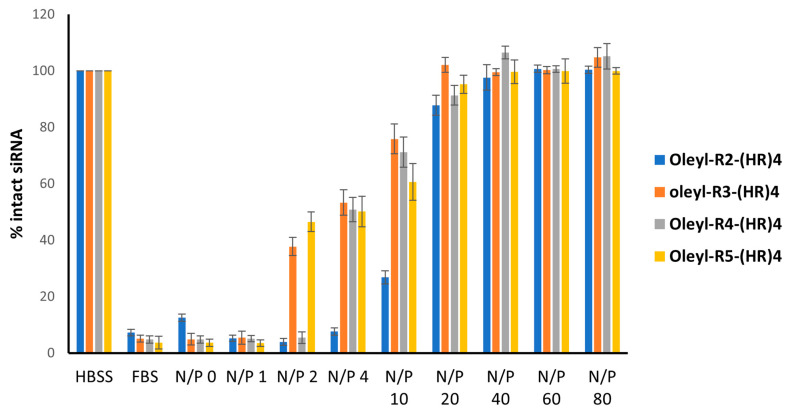
Gel retardation assay of oleyl-conjugated peptides/siRNA complexes after 24 h exposure to 25% *v/v* FBS and dissociation of complexes using heparin competition assay at N/P ratios ranging from 0 to 80. The bars indicate the mean intensity of the bands, representing percent siRNA bound to the peptides, quantified by Image Lab software. Error bars show the standard deviation (*n* = 3).

One of the essential characteristics of any promising siRNA delivery vehicle is its ability to protect the siRNA from the harsh serum environment containing nucleases. Serum stability assay was performed to evaluate the ability of the oleyl-conjugated peptides to protect the siRNA when exposed to 25% *v/v* FBS for 24 h. The representative images of the gels are shown in Figure 7. The gel images were quantified using Image Lab software; the data is shown as percent intact siRNA on the y-axis and oleyl-conjugated peptides on the x-axis. (Figure 8). One hundred percent intact siRNA can be seen in the HBSS solution, where almost all the siRNA was degraded in the presence of FBS. As the N/P ratio of the complexes is increased, the quantity of intact siRNA also increases, indicating that the oleyl conjugated peptides protected the siRNA from degradation by the serum. All of the conjugates show ~100% protection to siRNA at N/P 20 and above. This concludes that oleyl-conjugated peptides bind to siRNA significantly via ionic interactions and protect the siRNA from the serum enzymes and hence can improve the half-life of siRNA. 

### 3.8. STAT-3 Silencing Using Oleyl Conjugated Peptides/siRNA Complexes in MDA-MB-231 Cells

STAT3 is an essential member of the STAT family, a latent transcription factor that acts as an oncogene in several malignant diseases [58]. Several investigators reported that STAT3 aberrant expression was significantly associated with the status of lymph node metastasis in various malignant diseases [42,59,60,61]. Several STAT-3 siRNA delivery agents have been investigated but failed to reach clinical trials because of the cytotoxicity issue [62,63,64]. Very few studies have used a peptide-based delivery system for siRNA; therefore, we chose STAT-3 as a target protein using the novel fatty acyl conjugated histidine–arginine-based cell-penetrating peptide. We used the Western blotting technique to determine the expression of STAT-3 in MDA-MB-231 cells according to the method described in Section 2.10. Nontreated cells were used as a negative control, whereas the commercial transfection agent Lipofectamine 2000 was used as a positive control. It is pertinent to mention here that we also used scrambled siRNA treated cells as negative control and found no significant difference in STAT-3 expression between nontreated and scrambled siRNA treated cells (data not shown). A similar pattern was observed in flow cytometry and confocal microscopy assay, where we did not observe any significant difference in cellular uptake between Alexa-488 siRNA treated cells and nontreated cells. The lack of cellular uptake in case of free siRNA is attributed to its membrane impermeability due to the presence of negatively charged phosphate groups. GAPDH protein served as a housekeeping protein. The final siRNA concentration in each treatment group was 50 nM. The peptide concentrations in the complexes are described in Table 4, which are way less than the cytotoxic concentrations as determined by the cytotoxicity assay described in Section 3.2.

**Figure 9:**
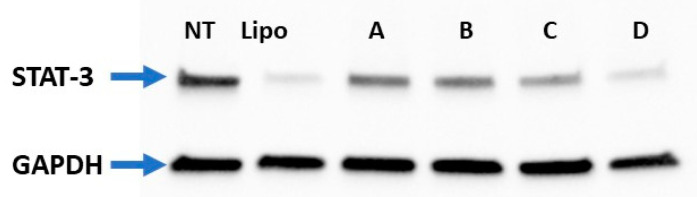
Western blot image of STAT-3 protein silencing in MDA-MB-231 cells. Where GAPDH served as a house-keeping protein; [NT = Nontreated Cells, Lipo= Lipofectamine 2000, A = Oleyl-R_2_-(HR)_4_, B = Oleyl-R_3_-(HR)_4_, C = Oleyl-R_4_-(HR)_4_, D = Oleyl-R_5_-(HR)_4_].

The representative Western blot image (Figure 9) shows the decreased expression of STAT-3 with an increase in the number of arginine residues in oleyl-conjugated peptides. For instance, oleyl-R_2_-(HR)_4_ caused ~68% of STAT-3 expression, oleyl-R_3_-(HR)_4_ showed ~55%, oleyl-R_4_-(HR)_4_ showed ~41% and Oleyl-R_5_-(HR)_4_ showed ~20% of the STAT-3 expression. The Western blotting data correlates with cellular internalization (Section 3.5).

**Figure 10:**
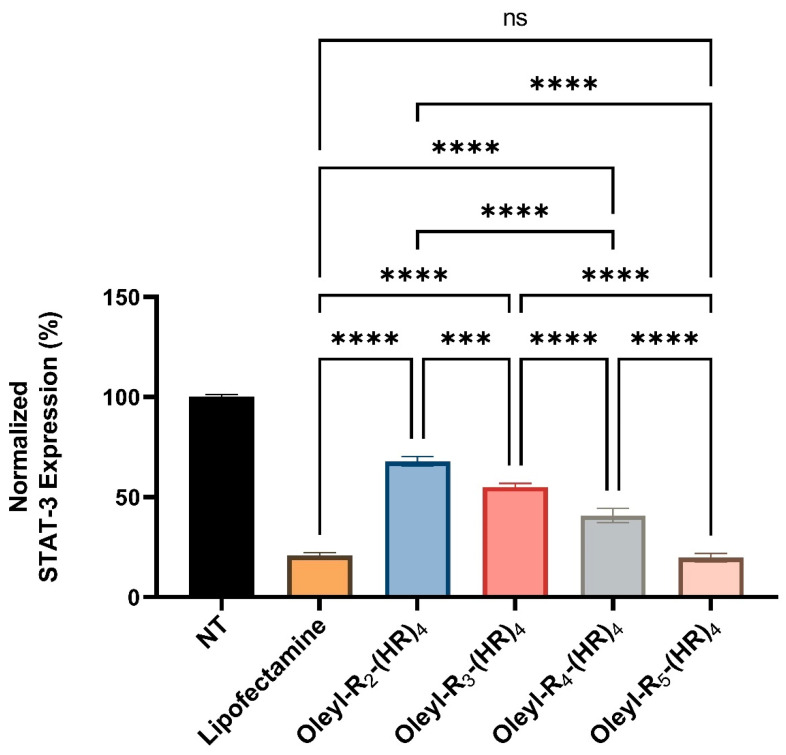
Normalized STAT-3 expression quantified from the Western blot after 48 h of treatment with oleyl conjugated peptides-siRNA complexes at N/P 40. ns means nonsignificant, *** indicates a *p*-value < 0.001, and **** indicate a *p*-value < 0.0001.

Furthermore, we quantified the Western blot data from three independent experiments using Image Lab software presented in Figure 10. The data indicates a comparable inhibition of STAT-3 expression with oleyl-R_5_-(HR)_4_ to that of lipofectamine. Another exciting trend is that increasing the number of arginine residues affects the delivery efficiency of the fatty acyl peptide conjugates which correlate with silencing of STAT-3 gene. For instance, there is a significant reduction in STAT-3 expression when the conjugates are compared with each other with the maximum reduction observed with the conjugate containing a total of nine arginine residues (oleyl-R_5_-(HR)_4_).

## 4. Conclusions

A series of oleyl-R_n_-(HR)_4_ conjugated peptides were synthesized, characterized, and purified. They were found noncytotoxic up to a concentration of ~20 µM in MCF-7, MCF-10A, and MDA-MB-231 cells. Oleyl-R_3_-(HR)_4_ and oleyl-R_4_-(HR)_4_ showed ~80-fold increase in cellular uptake of siRNA compared to free siRNA. Oleyl conjugated peptides formed nanocomplexes with siRNA, possibly due to the presence of fatty acyl chain, and showed appropriate zeta potential values required for interaction with the cell membranes. Both the visual confocal images as well as the quantitative flow cytometry date showed the significant siRNA cellular uptake using oleyl conjugated histidine–arginine cell penetrating peptides. The conjugates exhibited significant siRNA binding affinity and helped protect the siRNA from serum enzymes for up to 24 h at N/P ratio ≥ 20. STAT-3 protein was used as a model protein to investigate the effectiveness of the conjugated peptides for siRNA delivery. There appears a pattern in silencing efficiency of the conjugates with variable arginine residues. For instance, oleyl-R_2_-(HR)_4_ showed ~68% reduction in STAT-3 expression, oleyl-R_3_-(HR)_4_ caused ~55%, oleyl-R_4_-(HR)_4_ showed ~41%, and oleyl-R_5_-(HR)_4_ showed ~80% reduction in STAT-3 expression. Moreover, STAT-3 inhibition observed with oleyl-R_5_-(HR)_4_ was comparable to the commercially available transfection agent Lipofectamine 2000. Significant inhibition of STAT-3 expression provides a proof-of-concept that oleyl-conjugated histidine arginine peptide can provide a promising strategy in the efficient delivery of siRNA with subsequent silencing of the target protein. Besides, the data from this research may provide a framework for determining the appropriate dose of the peptide-siRNA complex that can be subsequently employed for formulating a dosage form for in vivo studies to develop siRNA therapeutics. Exploration of fatty acyl histidine–arginine-containing CPPs provides a promising approach to siRNA delivery.

## Figures and Tables

**Table 1:** MALDI-TOF characterization of synthesized oleyl conjugated peptides.

Sr. No	Conjugate	Sequence	Chemical Formula	Exact Mass	Found (M/Z)
1	Oleyl-(HR)_4_	Oleyl-(HR)_4_-OH	C_66_H_110_N_28_O_10_	1454.896	1455.996 [M + H]^+^
2	Oleyl-R_1_-(HR)_4_	Oleyl-R-(HR)_4_-OH	C_72_H_122_N_32_O_11_	1610.997	1612.230 [M + 2 H]^+^
3	Oleyl-R_2_-(HR)_4_	Oleyl-RR-(HR)_4_-OH	C_78_H_134_N_36_O_12_	1767.098	1767.642 [M]^+^
4	Oleyl-R_3_-(HR)_4_	Oleyl-RRR-(HR)_4_-OH	C_84_H_146_N_40_O_13_	1923.199	1925.019 [M + 2 H]^+^
5	Oleyl-R_4_-(HR)_4_	Oleyl-RRRR-(HR)_4_-OH	C_90_H_158_N_44_O_14_	2079.300	2080.422 [M + H]^+^
6	Oleyl-R_5_-(HR)_4_	Oleyl-RRRRR-(HR)_4_-OH	C_96_H_171_N_49_O_14_	2234.417	2236.281 [M + 2 H]^+^

**Table 2:** Relation of N/P with peptide concentration and amount of ionizable nitrogen in the peptide conjugates.

Peptide	N/P	siRNA (µM)	Peptide Conc. (µM)	W/W Ratio (Peptide/siRNA)	Molar Ratio (Peptide/siRNA)
Oleyl-(HR)_4_	40	1	43.6	52.5	480
Oleyl-R_1_-(HR)_4_	40	1	34.9	46.5	384
Oleyl-R_2_-(HR)_4_	40	1	29.1	42.5	320
Oleyl-R_3_-(HR)_4_	40	1	24.9	39.7	274
Oleyl-R_4_-(HR)_4_	40	1	21.8	37.5	240
Oleyl-R_5_-(HR)_4_	40	1	19.4	35.9	213

**Table 3:** Hydrodynamic diameter of oleyl-conjugated peptides/siRNA complexes.

Peptide-siRNA Complex at N/P 40.	Z-Ave (d.nm)	SD	PDI	SD
Oleyl-(HR)_4_	120.1	±1.3	0.213	±0.03
Oleyl-R_1_-(HR)_4_	116.9	±2.5	0.201	±0.06
Oleyl-R_2_-(HR)_4_	115.6	±1.6	0.217	±0.05
Oleyl-R_3_-(HR)_4_	117	±1.8	0.229	±0.03
Oleyl-R_4_-(HR)_4_	115.2	±2.3	0.237	±0.07
Oleyl-R_5_-(HR)_4_	115.6	±2.1	0.213	±0.04

**Table 4:** Treatment concentrations of Peptides for Western blotting assay.

Compound	N/P Ratio	Final Concentration of Peptide in the Complex (µM)	W/W Ratio (Peptide/siRNA)	Molar Ratio (Peptide/siRNA)
Oleyl-R_2_-(HR)_4_	40	16	42.51	320
Oleyl-R_3_-(HR)_4_	40	13.71	39.66	274.29
Oleyl-R_4_-(HR)_4_	40	12	37.53	240
Oleyl-R_5_-(HR_)4_	40	10.67	35.87	213.33

## Data Availability

Not applicable.

## Supplementary Materials

The following supporting information can be downloaded at: https://www.mdpi.com/article/10.3390/pharmaceutics14040881/s1, consisting of MALDI-TOF mass spectrometry spectrum (Figures S7–S12) and analytical HPLC chromatograms (Figures S1–S6).
