# Supplementary material for: Oleyl Conjugated Histidine-Arginine Cell-Penetrating Peptides as Promising Agents for siRNA Delivery
Source: Pharmaceutics. 2022 Apr 18;14(4):881. doi: 10.3390/pharmaceutics14040881 (PMC9028392; doi:10.3390/pharmaceutics14040881)
Supplement: Supplementary file 1 [file pharmaceutics-14-00881-s001.zip › pharmaceutics-1637276-supplementary.pdf]

pharmaceutics

MDPI

Supplementary Materials: Oleyl Conjugated Histidine-Arginine Cell-Penetrating Peptides as Promising Agents for siRNA Delivery

Muhammad Imran Sajid, Dindyal Mandal, Nagla Salem El-Sayed, Sandeep Lohan, Jonathan Moreno, and Rakesh Kumar Tiwari

Contents

1. Analytical HPLC chromatogram of conjugated peptides Pages 2 - 5
2. MALDI-TOF mass spectrum of conjugated peptides Pages 6 - 11

1. Analytical HPLC chromatogram of conjugated peptides

HPLC Method: The purity of the peptides was determined using reverse phase analytical HPLC method at a flow rate 0.5 mL/min on Shimadzu (LC-20ADXR) with a gradient system of water with 0.1% TFA (solvent A) and mixture of acetonitrile and isopropyl alcohol (8:2) with 0.1% TFA (solvent B), using (Phenomenex Luna, 4 µm C18 150 × 4.6 mm HPLC Column).

Time	% Solvent B
0.01	5
5.0	5
10.0	12
25.0	60
27.0	95
32.0	95
32.01	5
38.0	5

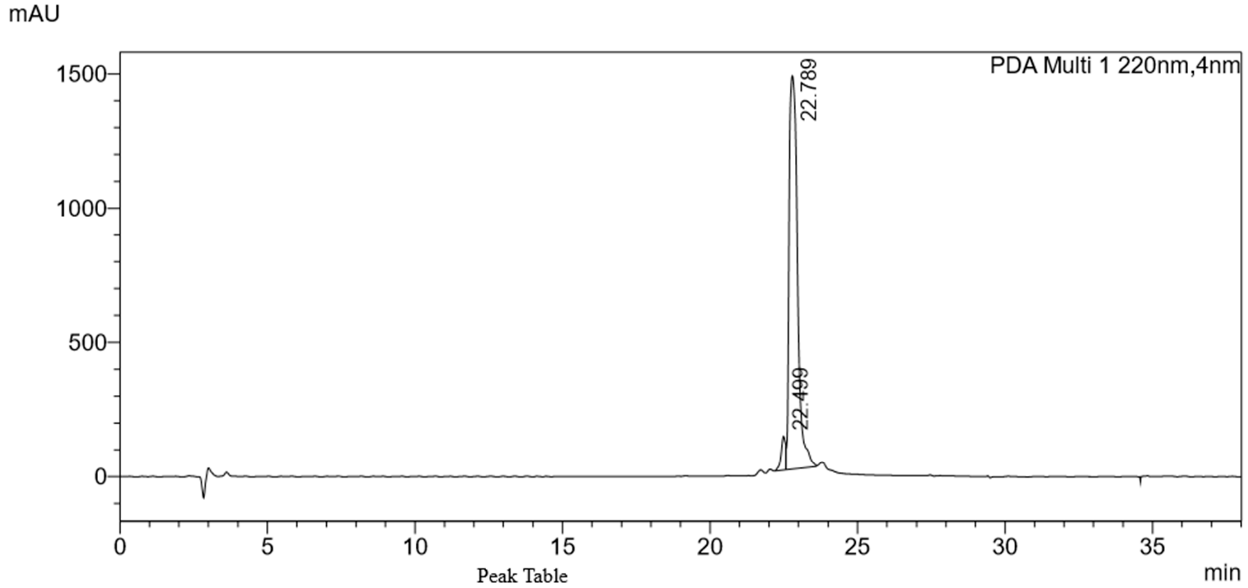

Peak Table

PDA Ch1 220nm

Peak#	Ret. Time	Area	Height	Area%
1	22.499	1266444	126579	4.149
2	22.789	29261067	1464350	95.851
Total		30527511	1590929	100.000

Figure S1. HPLC chromatogram of Oleyl-(HR)$_4$.

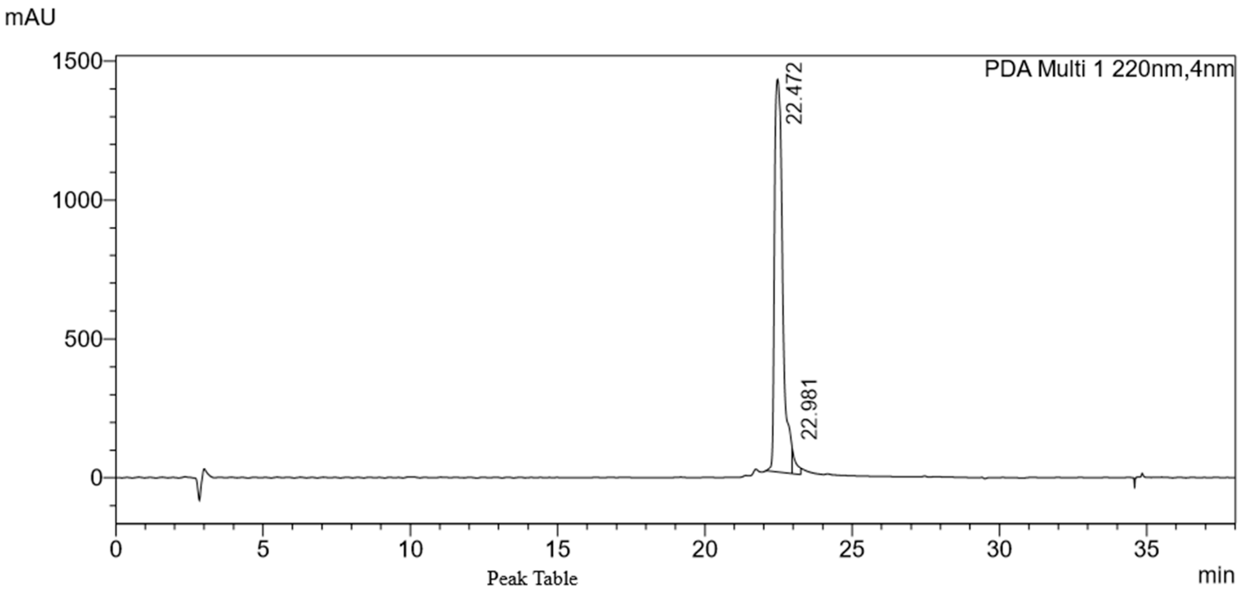

Peak Table

PDA Ch1 220nm

Peak#	Ret. Time	Area	Height	Area%
1	22.472	26733246	1414071	97.279
2	22.981	747886	86240	2.721
Total		27481131	1500311	100.000

Figure S2. HPLC chromatogram of Oleyl-R_1-$(HR)_4$.

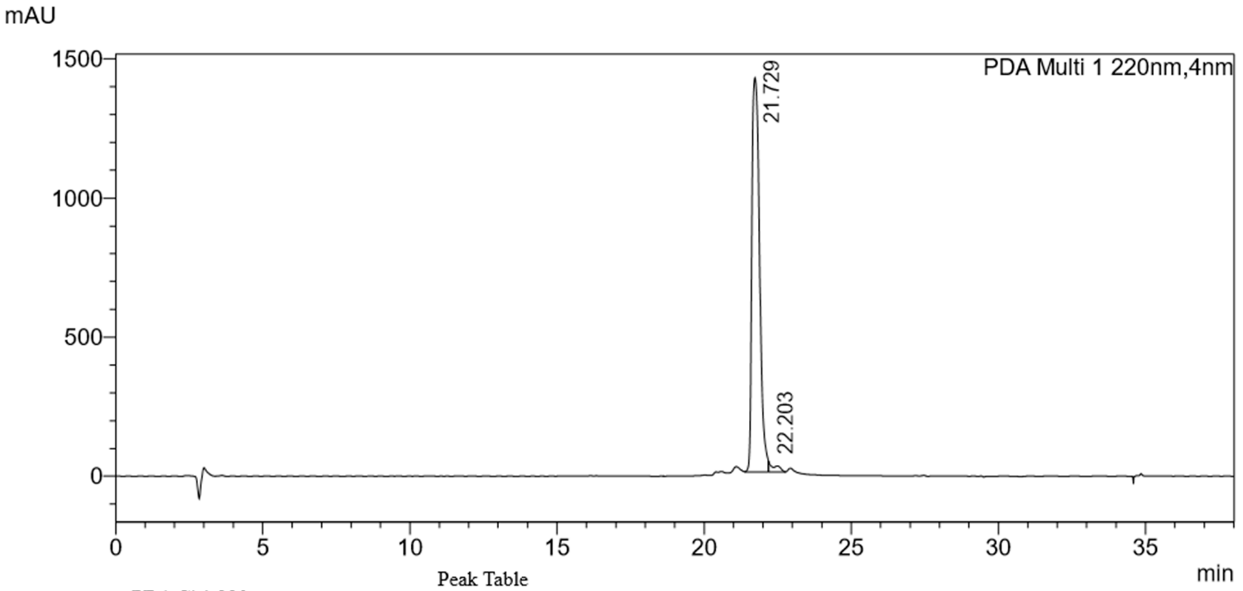

Peak Table

PDA Ch1 220nm

Peak#	Ret. Time	Area	Height	Area%
1	21.729	25817284	1417203	97.862
2	22.203	564034	37931	2.138
Total		26381318	1455134	100.000

Figure S3. HPLC chromatogram of Oleyl-R_2-$(HR)_4$.

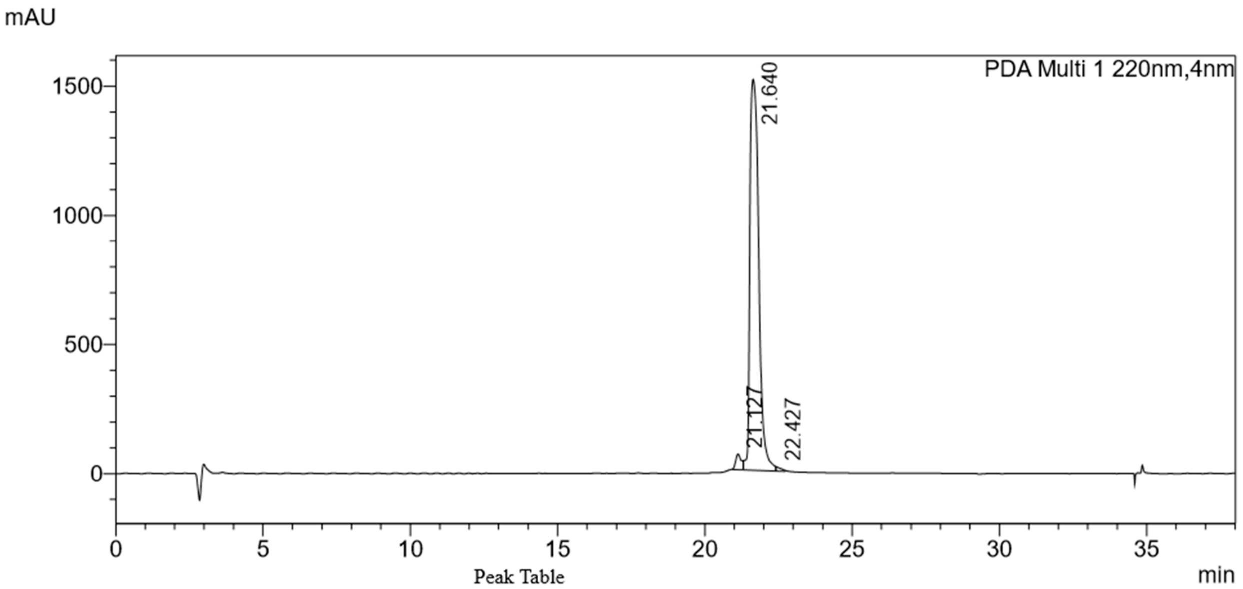

Peak Table

PDA Ch1 220nm

Peak#	Ret. Time	Area	Height	Area%
1	21.127	813311	61289	2.469
2	21.640	31937009	1511905	96.965
3	22.427	186481	16750	0.566
Total		32936802	1589945	100.000

Figure S4. HPLC chromatogram of Oleyl-R_3-$(HR)_4$.

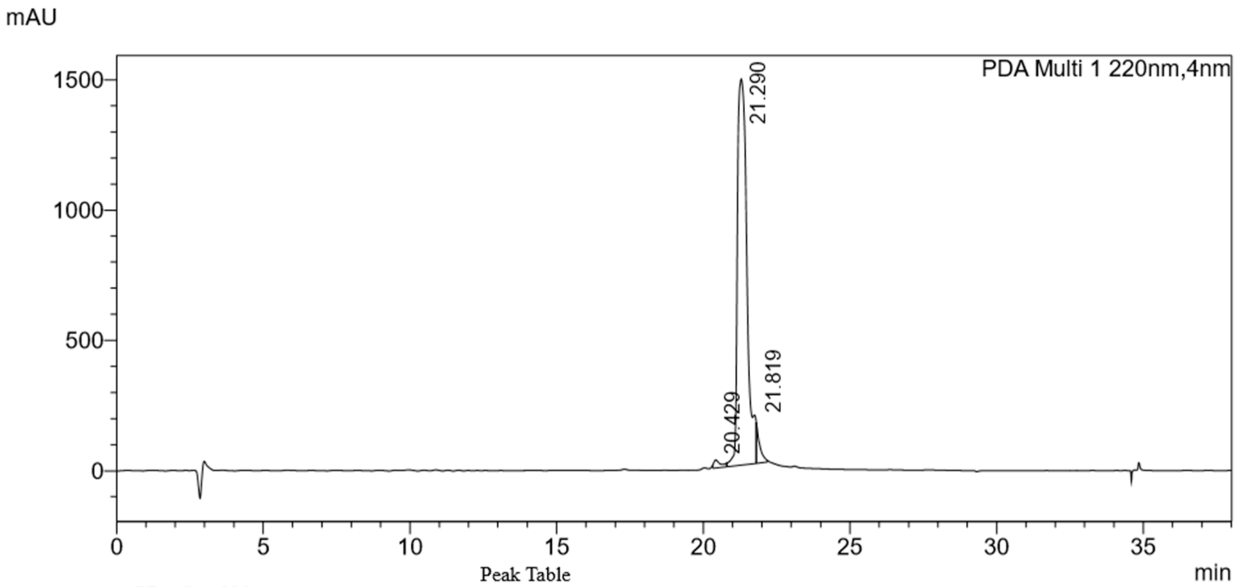

Peak Table

PDA Ch1 220nm

Peak#	Ret. Time	Area	Height	Area%
1	20.429	516503	30373	1.500
2	21.290	32812289	1480141	95.298
3	21.819	1102489	155848	3.202
Total		34431280	1666362	100.000

Figure S5. HPLC chromatogram of Oleyl-R_4-$(HR)_4$.

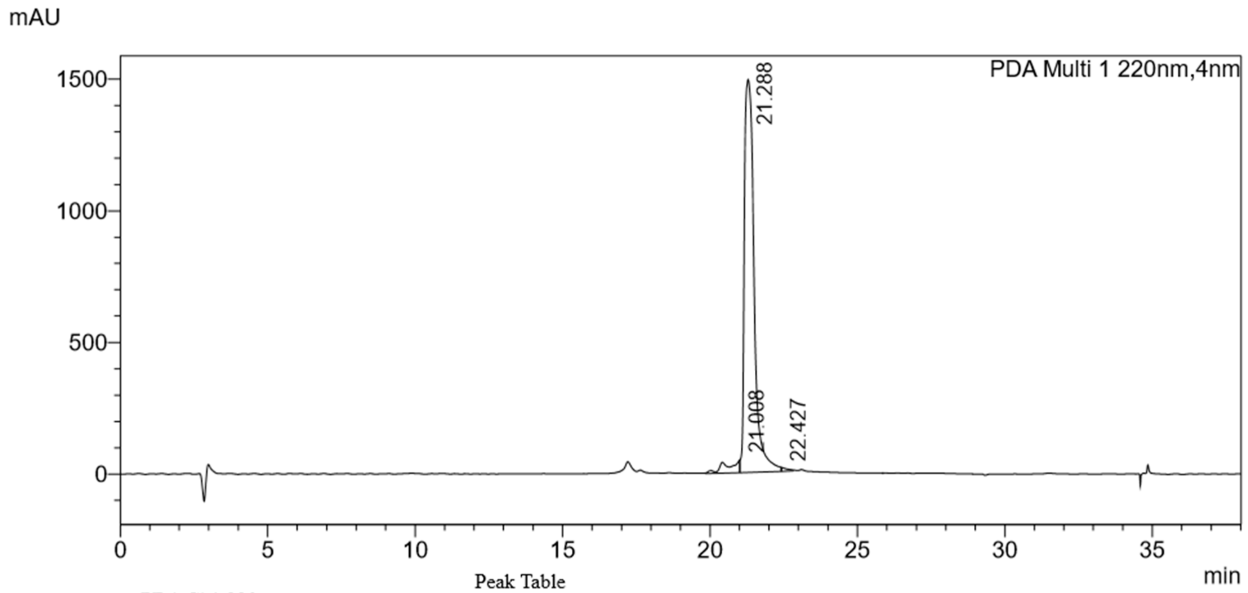

Peak Table

PDA Ch1 220nm

Peak#	Ret. Time	Area	Height	Area%
1	21.008	1537026	47432	4.340
2	21.288	33680178	1490856	95.104
3	22.427	196676	15148	0.555
Total		35413880	1553436	100.000

Figure S6. HPLC chromatogram of Oleyl-R_5-$(HR)_4$.

2. **MALDI-TOF mass spectrum of conjugated peptides**
3.

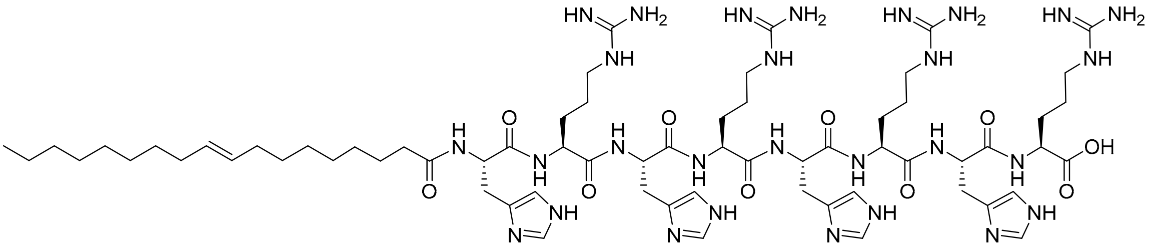

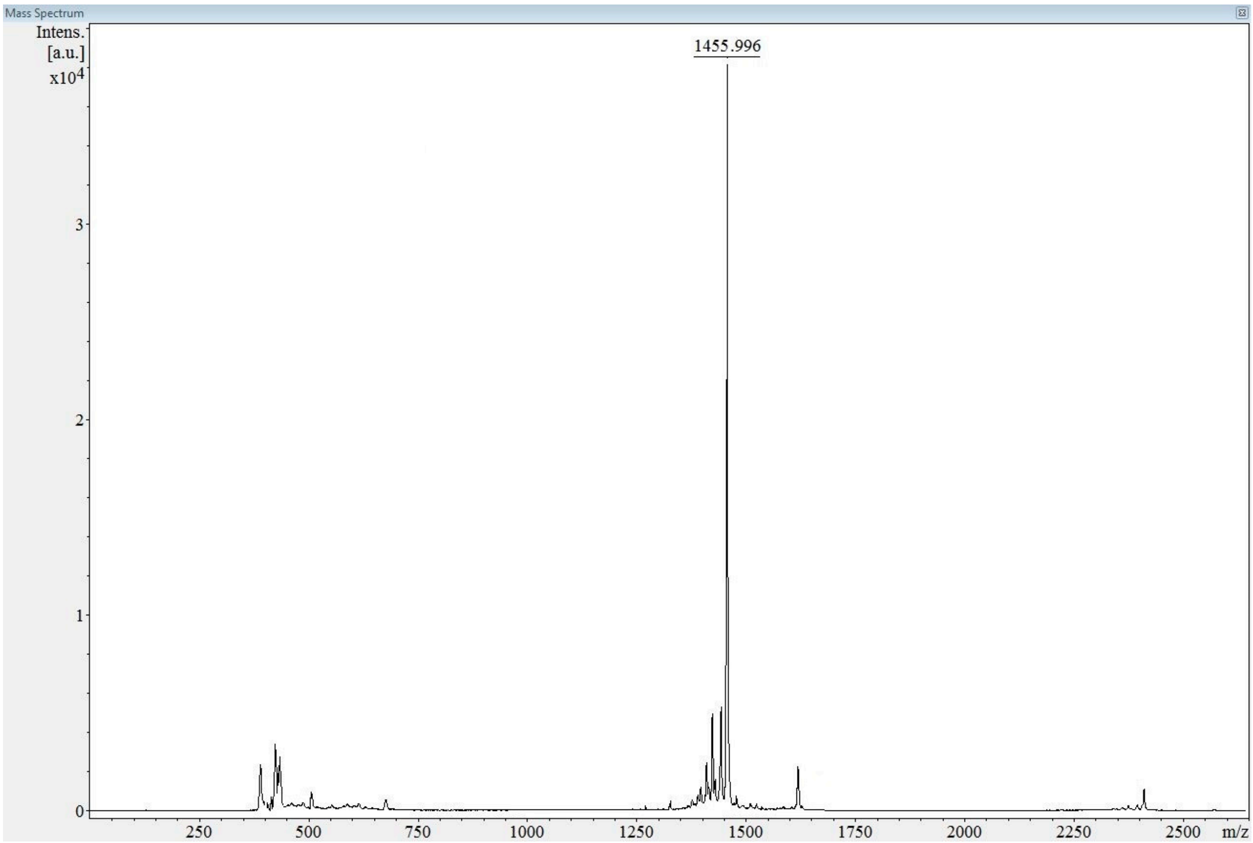

Figure S7. MALDI-TOF mass spectrum of Oleyl-$(HR)_4$.

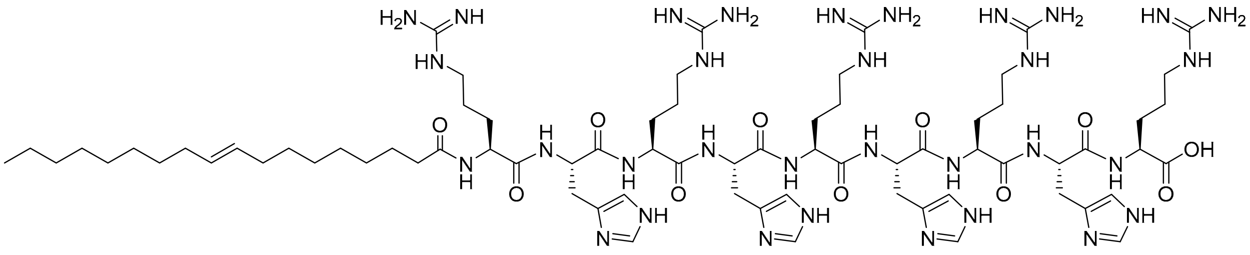

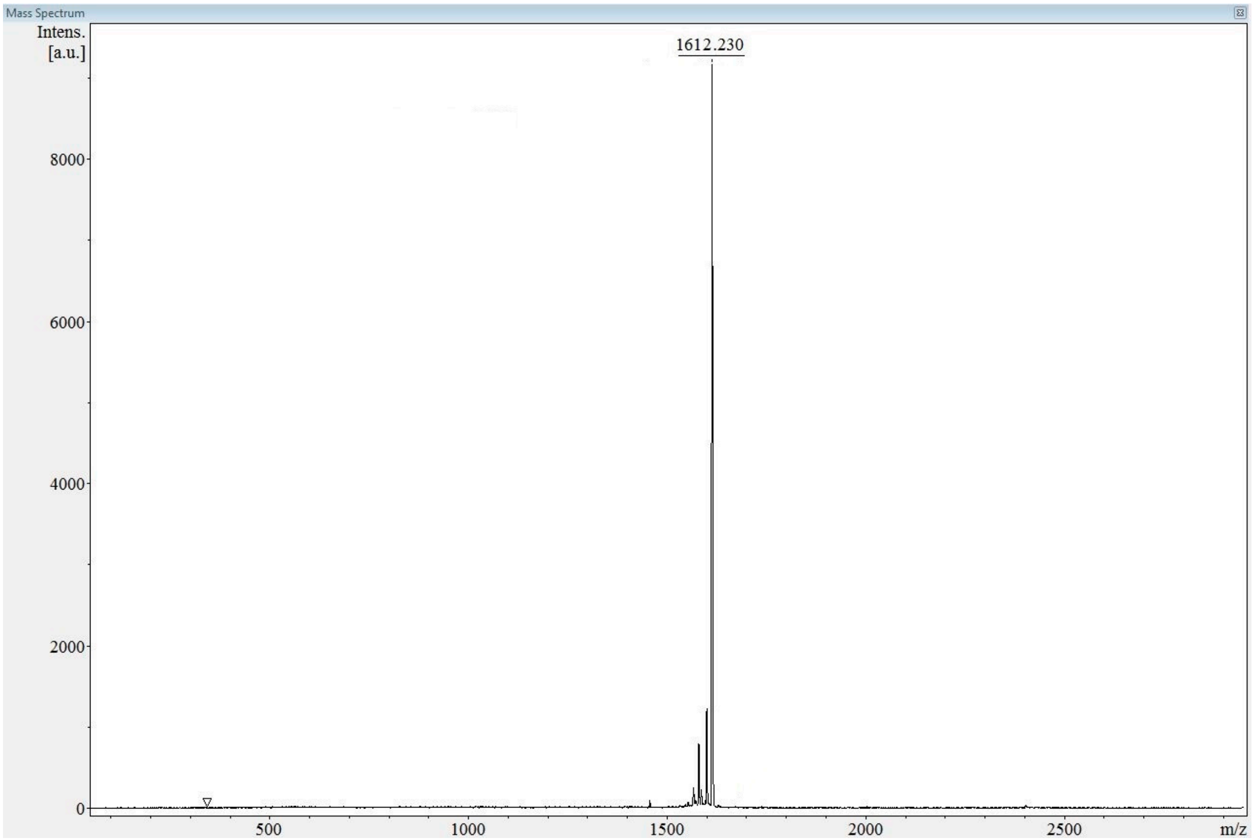

Figure S8. MALDI-TOF mass spectrum of Oleyl-R_1-$(HR)_4$.

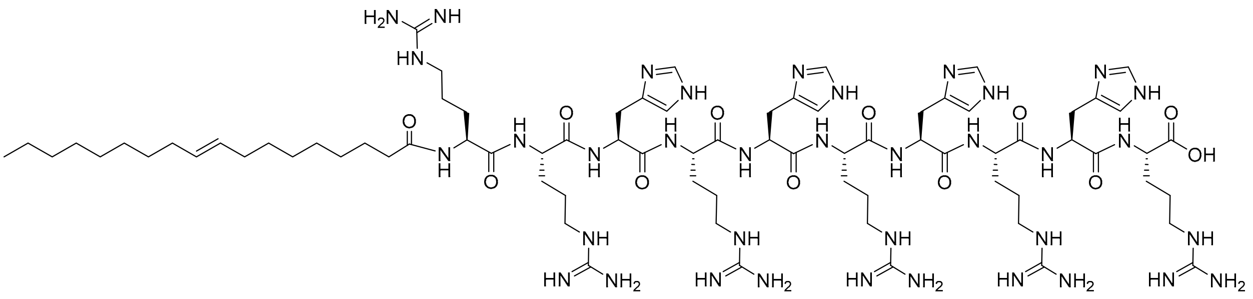

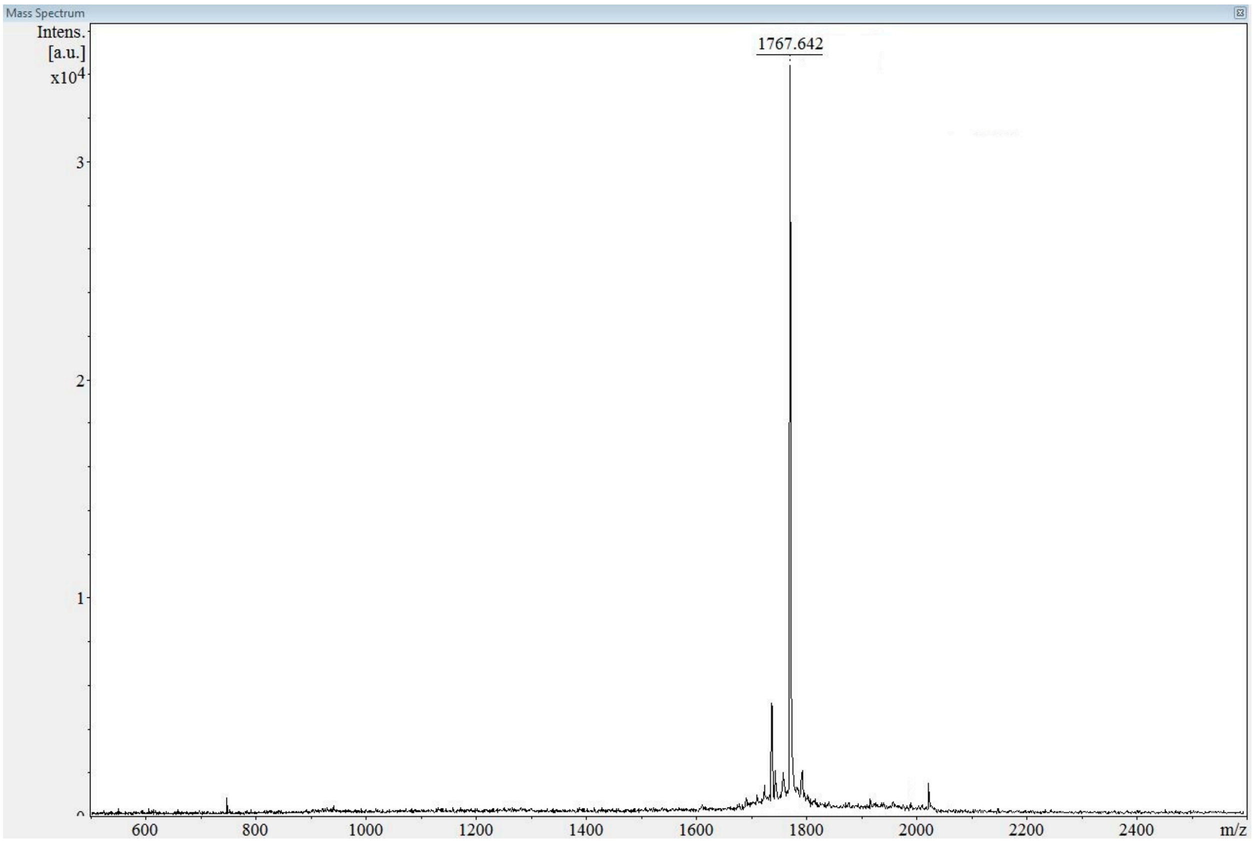

Figure S9. MALDI-TOF mass spectrum of Oleyl-R_2-$(HR)_4$.

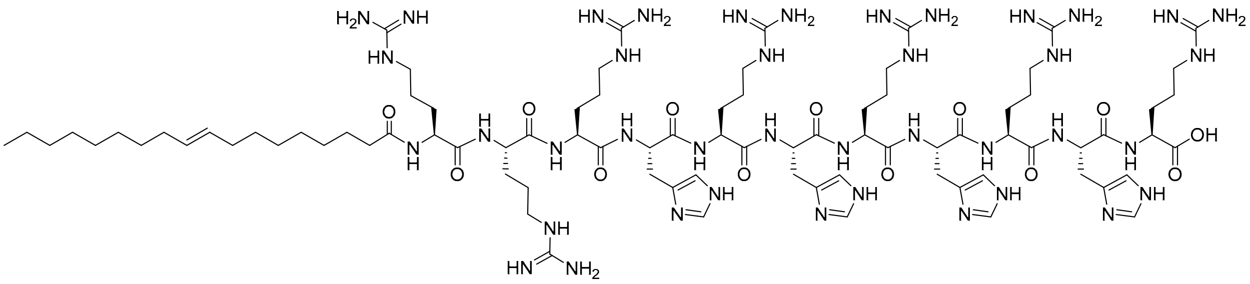

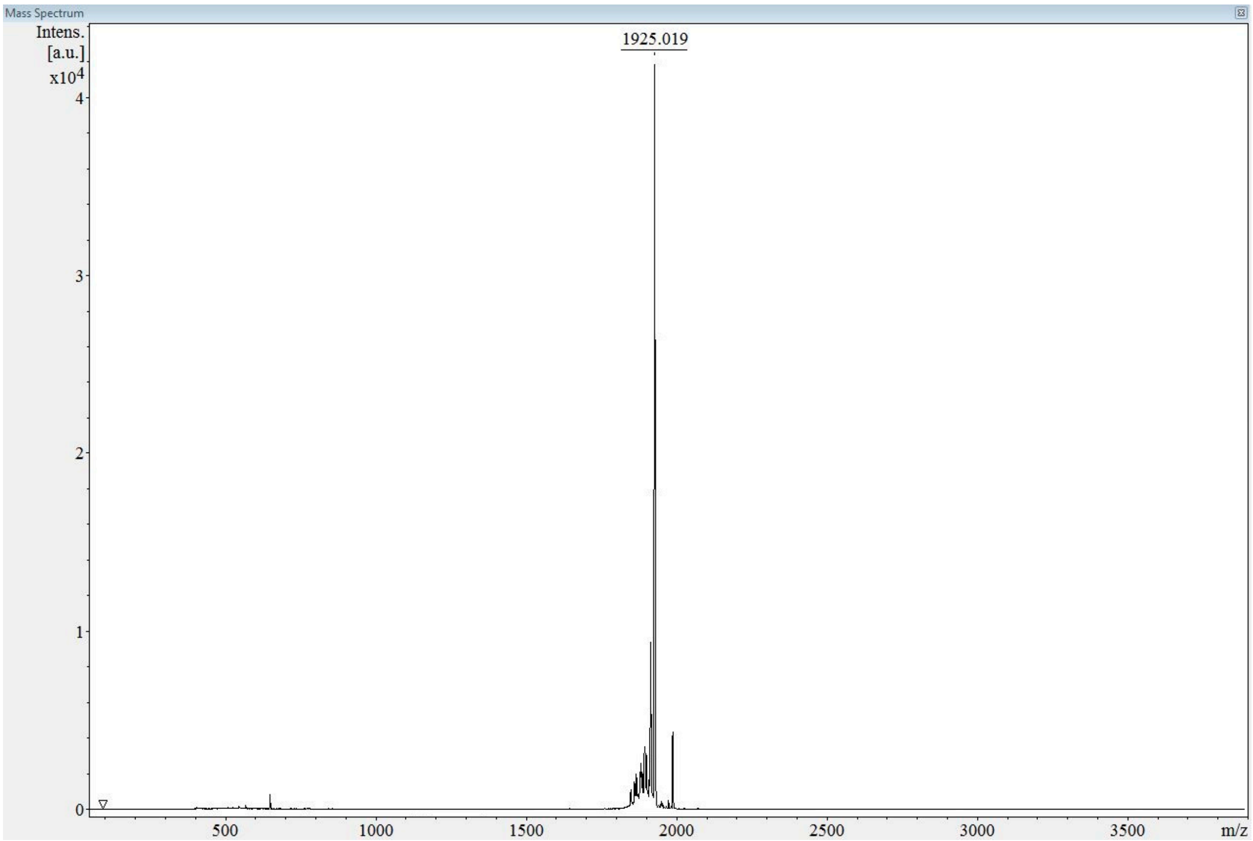

Figure S10. MALDI-TOF mass spectrum of Oleyl-R3-(HR)4.

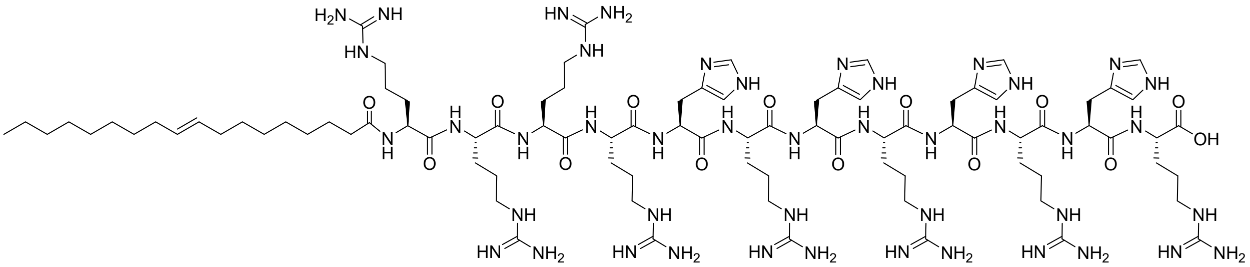

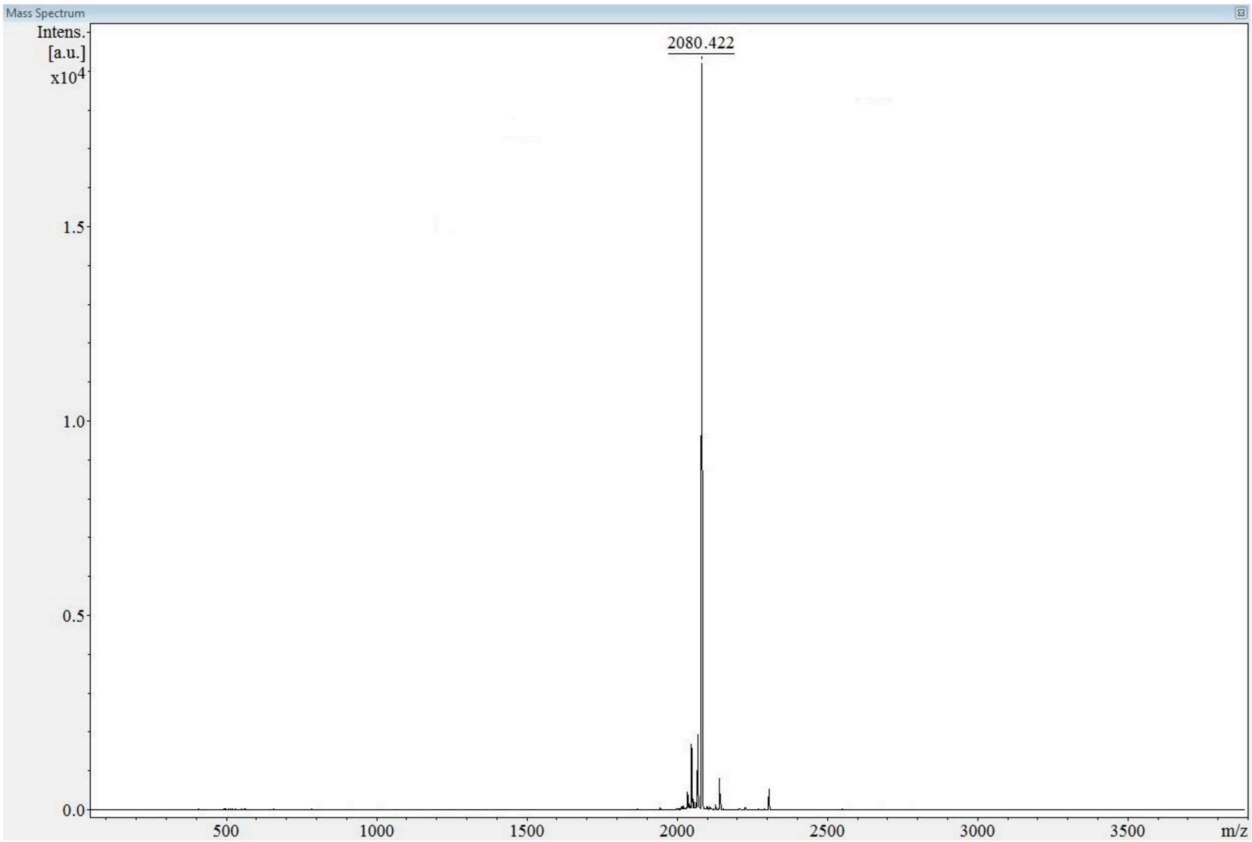

Figure S11. MALDI-TOF mass spectrum of Oleyl-R_4-$(HR)_4$.

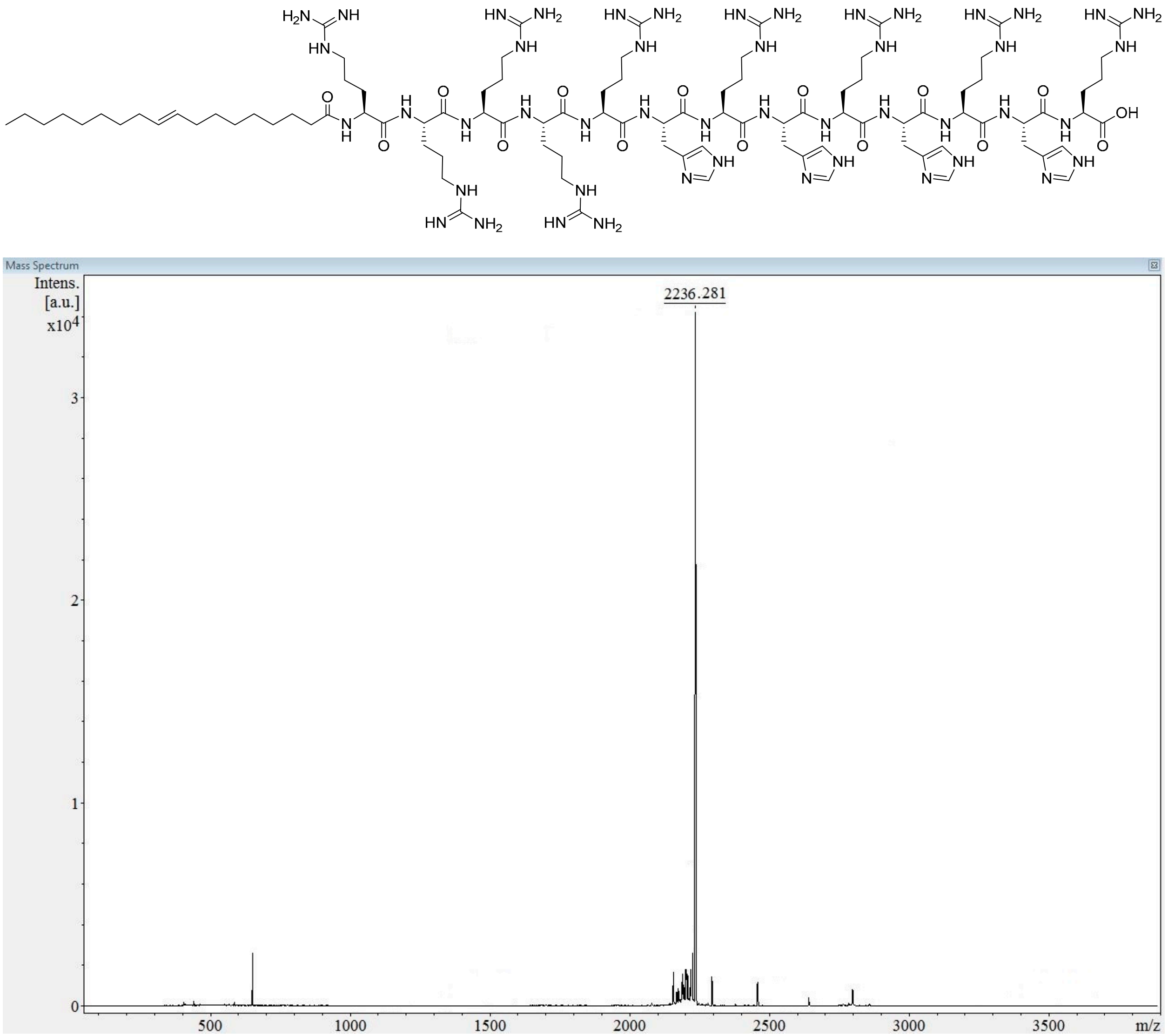

Figure S12. MALDI-TOF mass spectrum of Oleyl-R_5-$(HR)_4$.
